# The UFM1 Pathway Impacts HCMV US2-Mediated Degradation of HLA Class I

**DOI:** 10.3390/molecules26020287

**Published:** 2021-01-08

**Authors:** A.B.C. Schuren, I.G.J. Boer, E.M. Bouma, M.L. Van de Weijer, A.I. Costa, P. Hubel, A. Pichlmair, R.J. Lebbink, E.J.H.J. Wiertz

**Affiliations:** 1 Department of Medical Microbiology, University Medical Center Utrecht, Postbus 85500, 3508 GA Utrecht, The Netherlands; A.B.C.Schuren-2@umcutrecht.nl (A.B.C.S.); G.J.Boer@umcutrecht.nl (I.G.J.B.); e.m.bouma@umcg.nl (E.M.B.); michael.vandeweijer@path.ox.ac.uk (M.L.v.d.W.); acorreia@umcutrecht.nl (A.I.C.); 2 Department of Medical Microbiology, University Medical Center Groningen, Postbus 30001, 9700 RB Groningen, The Netherlands; 3 Sir William Dunn School of Pathology, University of Oxford, Oxford OX1 3RE, UK; 4 Innate Immunity Laboratory, Max-Planck Institute for Biochemistry, Am Klopferspitz 18, Martinsried, D-82152 Munich, Germany; philipp.hubel@uni-hohenheim.de (P.H.); apichl@biochem.mpg.de (A.P.); 5 Core Facility Hohenheim, Universität Hohenheim, Emil-Wolff-Straße 12, D-70599 Stuttgart, Germany; 6 School of Medicine, Institute of Virology, Technical University of Munich, Schneckenburgerstr 8, D-81675 Munich, Germany; 7 German Center for Infection Research (DZIF), Munich Partner Site, D-85764 Neuherberg, Germany

**Keywords:** ER-associated protein degradation (ERAD), dislocation, HCMV protein US2, ubiquitin-fold modifier1 (UFM1), UFMylation, HLA class I

## Abstract

To prevent accumulation of misfolded proteins in the endoplasmic reticulum, chaperones perform quality control on newly translated proteins and redirect misfolded proteins to the cytosol for degradation by the ubiquitin-proteasome system. This pathway is called ER-associated protein degradation (ERAD). The human cytomegalovirus protein US2 induces accelerated ERAD of HLA class I molecules to prevent immune recognition of infected cells by CD8^+^ T cells. Using US2-mediated HLA-I degradation as a model for ERAD, we performed a genome-wide CRISPR/Cas9 library screen to identify novel cellular factors associated with ERAD. Besides the identification of known players such as TRC8, p97, and UBE2G2, the ubiquitin-fold modifier1 (UFM1) pathway was found to affect degradation of HLA-I. UFMylation is a post-translational modification resembling ubiquitination. Whereas we observe ubiquitination of HLA-I, no UFMylation was detected on HLA-I or several other proteins involved in degradation of HLA-I, suggesting that the UFM1 pathway impacts ERAD in a different manner than ubiquitin. Interference with the UFM1 pathway seems to specifically inhibit the ER-to-cytosol dislocation of HLA-I. In the absence of detectable UFMylation of HLA-I, UFM1 may contribute to US2-mediated HLA-I degradation by misdirecting protein sorting indirectly. Mass spectrometry analysis of US2-expressing cells showed that ribosomal proteins are a major class of proteins undergoing extensive UFMylation; the role of these changes in protein degradation may be indirect and remains to be established.

## 1. Introduction

When newly translated secretory proteins are inserted into the ER, quality control must occur to ensure that misfolded proteins do not accumulate and disturb ER function. In the ER, protein folding is continuously monitored by molecular chaperones. When a protein fails to acquire its correct conformation, it is transferred to the ER-associated protein degradation (ERAD) pathway. Substrates of ERAD are transported over the ER membrane towards the cytosol, where they are ubiquitinated and degraded by the proteasome. 

With over 70 diseases associated with ERAD [1,2], including cystic fibrosis and Parkinson’s disease, a better understanding of this protein degradation pathway is required. Because viruses depend on and manipulate their host cells, they provide useful models to study a wide range of cellular processes. Indeed, many viruses exploit ERAD to facilitate virus replication [3] or to evade immune recognition [4,5]. These manipulation strategies can be exploited to study protein degradation. 

Human cytomegalovirus (HCMV) is a herpesvirus that causes severe congenital defects when it infects pregnant women [6]. The virus can successfully evade the immune system, allowing it to persist in the body lifelong. HCMV induces accelerated ERAD of HLA class I molecules to prevent recognition of virus-infected cells by CD8^+^ T lymphocytes. The viral proteins responsible for this, US2 and US11, serve as important models to study the degradation of ER-resident proteins. These HCMV proteins have allowed the identification of many key mammalian ERAD factors, including Derlins, VIMP [7,8], and the ubiquitin E3 ligases TRC8 [9] and TMEM129 [10,11]. 

Despite the identification of a number of factors involved in ERAD, many questions remain to be answered. It is thought that the protein complexes required for ERAD are (partially) specific to the substrate that is degraded, in combination with some general ERAD players such as p97/VCP and the proteasome. While the ubiquitination machinery for US2-mediated degradation of HLA class I has been identified [9,10,11,12,13], knowledge about other ERAD factors is lacking.

Here, we performed a genome-wide CRISPR/Cas9 library screen to identify novel host genes involved in US2-mediated HLA class I degradation. Notably, besides known ERAD-related factors, we identified all known factors of the UFMylation pathway to affect HLA-I degradation via US2. UFMylation is a ubiquitin-like post-translational modification that involves covalent attachment of ubiquitin-fold modifier 1 (UFM1), a ~9.1 kDA protein, to target proteins. Under the experimental conditions used, however, we did not detect direct UFMylation of HLA-I or other proteins involved in ERAD. A mass spectrometry analysis showed that UFMylation predominantly occurs on ribosomal proteins in US2-expressing cells. We therefore speculate that UFM1 may affect US2-mediated protein degradation indirectly, via a mechanism potentially involving the ribosome.

## 2. Results

### 2.1. A Genome-Wide CRISPR/Cas9 Library Screen Identifies the UFM1 Pathway to Impact HCMV US2-Mediated ERAD

**Figure 1:**
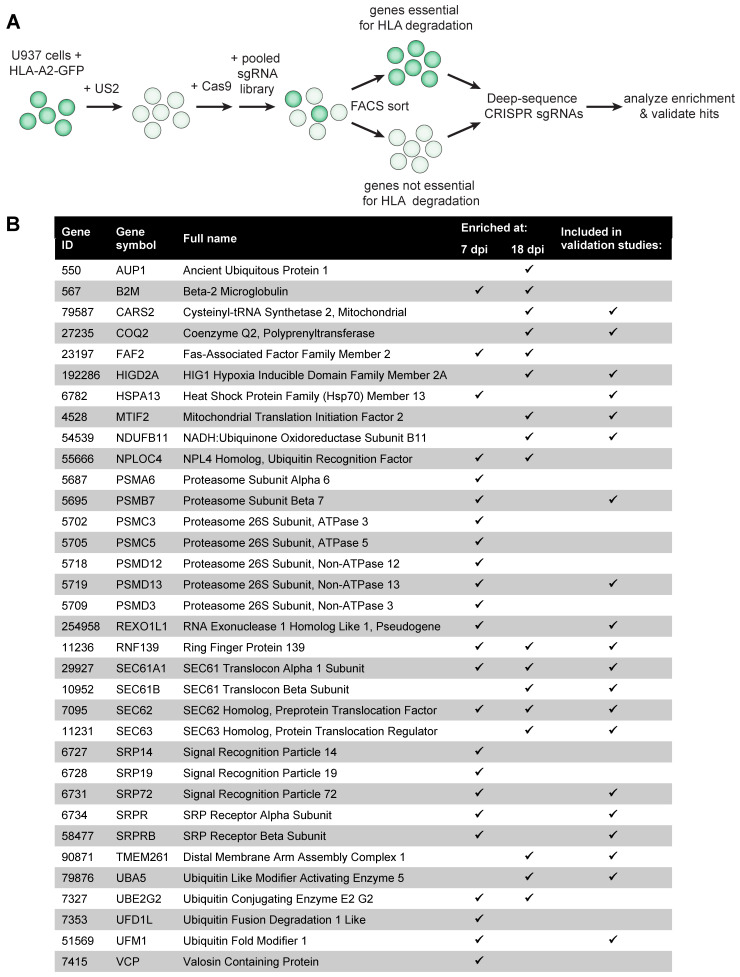

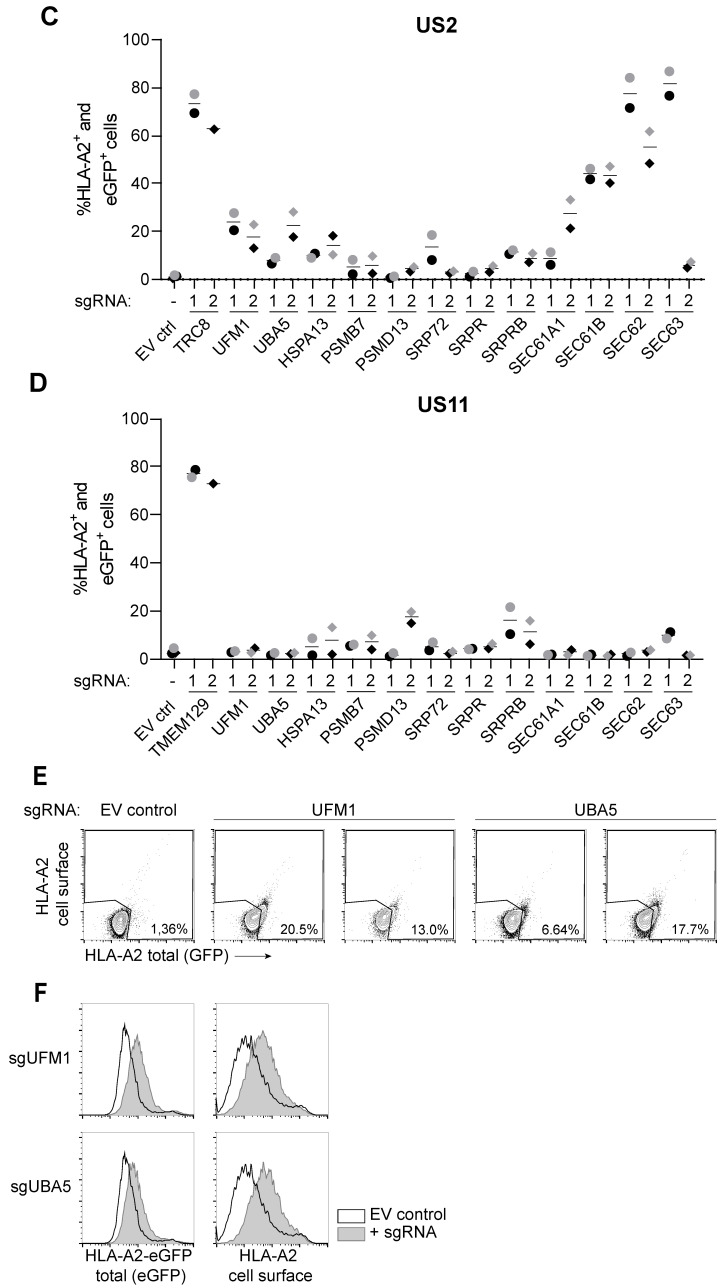
A genome-wide CRISPR/Cas9 library screen identifies the UFM1 pathway to affect HCMV US2-mediated degradation of HLA class I. (**A**) Schematic overview of the genome-wide CRISPR/Cas9 library screen setup. (**B**) Overview of hits identified in the library screen and genes selected for validation studies. Flow cytometric analysis for the library screen was performed at 7- and 18 days post-lentiviral transduction with the sgRNA library. The checkmarks indicate at which timepoint the respective genes were identified. (**C**) Validation of the screen at 14 days post-transduction of the sgRNAs. HLA-A2-eGFP U937 cells co-expressing US2 and SpCas9 were transduced with single sgRNAs targeting the presented genes. For each gene, the two most enriched sgRNAs from the screen were validated. The ubiquitin E3 ligase TRC8 was included as positive control, and an empty vector (EV ctrl) was included as negative control. Rescue of HLA-A2-eGFP (as measured by assessing eGFP levels and cell surface staining with an HLA-A2-specific antibody) was measured by flow cytometry. Validation was performed twice at 7, 11, 14, 18, and 21/28 days post-infection. The other timepoints are shown in Figure S1. Circles (sgRNA1) and diamonds (sgRNA2) in black or grey represent two independent experiments. (**D**) Same as in (**C**), although US11-expressing U937 cells were tested, instead of US2-expressing cells. HLA-A2 rescue observed in the polyclonal knockout cells are specific to US2 for the majority of genes. TMEM129 was taken along as a positive control for these US11-expressing cells, as this ubiquitin ligase is essential for US11 function. (**E**) Flow cytometry dot plots of UBA5- and UFM1 sgRNA-targeted cells from the cells shown in Figure 1C. (**F**) UFM1- and UBA5-targeting sgRNAs were introduced in a different cell type (U373 cells), and rescue of HLA-A2-eGFP was measured by flow cytometry.

To identify novel human genes involved in HCMV US2-mediated degradation of ER-resident HLA class I molecules (HLA-I), we performed a genome-wide CRISPR/Cas9 library screen. U937 cells expressing a C-terminally eGFP-tagged HLA-A2 chimera, as well as HCMV US2 and Cas9, were transduced in duplicate with the GeCKO v2 CRISPR library, targeting 19,050 genes with 6 single guideRNAs (sgRNAs) per gene (Figure 1A). Depleting a gene that is crucial for US2-mediated HLA class I degradation is expected to rescue the eGFP-tagged HLA-A2 chimera from degradation, thereby increasing eGFP levels in the cell. We used FACS to select cells displaying enhanced levels of eGFP (rescued eGFP-tagged HLA-A2 expression), as well as PE (cell surface expression of HLA-A2 as detected by an antibody staining) at 7 and 18 days post-transduction. Cells displaying low eGFP- and HLA-A2 cell surface expression were sorted as a control. As ERAD is an important cellular pathway that is essential for cell viability, the 7-day timepoint was included to allow for the identification of ERAD factors that could also be critical for cell survival. We next sequenced the lentiviral sgRNA sequences from the eGFP^+^/HLA-A2^+^ and control cell populations by Illumina sequencing and used the MaGeCK package to compare the HLA-A2^+^ population *versus* the control at both timepoints. The overlap of the top 300 genes was used to select genes for further analysis (Figure 1B). As expected, genes essential for cell survival (such as proteasome subunits) were identified only at the early timepoint, whereas others, such as AUP1 and SEC63, were seen only at the 18-day timepoint. TRC8 (RNF139), the known ubiquitin E3 ligase for US2-mediated HLA-I degradation [9], was our main hit at both timepoints, and therefore used as positive control for the following studies. We also identified other known players in US2-mediated ERAD, such as the E2 enzyme UBE2G2 [13], p97/VCP [14], various proteasomal subunits [15] and multiple members of the SEC61 complex [15]. The role of the SEC61/62/63 complex in US2-mediated HLA-I degradation is studied in more detail in [16] (preprint). We also identified multiple components of the signal recognition particle, which may be related to US2 translocation [16] (preprint). Other hits in this screen have been described in the context of ERAD previously, but not specifically for degradation of HLA-I by US2: the UBE2G2-binding ER-membrane protein AUP1 [17], FAF2/UBXD8 [18], and the p97 co-factors Npl4 and Ufd1 [14]. We previously showed that Npl4 and Ufd1 do play a role in US2-mediated HLA-I degradation in U937 cells [16] (preprint). A selection of the hits from both timepoints (Figure 1B, see M&M for details) was subjected to further validation (Figure 1C). To do so, the two most enriched sgRNAs per gene were selected from the library and were individually transduced into U937 cells co-expressing HLA-A2-eGFP, US2 and Cas9. HLA-A2-eGFP expression was assessed by flow cytometry at 7, 11, 14, 18 and 28 days post-transduction (Figure 1C and Figure S1). At 14 d.p.i. hits from both the early and the late timepoint can be observed (Figure 1C). Most, but not all, sgRNAs showed enhanced eGFP expression in a subset of transduced cells, confirming that these were bona-fide hits in the screen and regulate HLA-A2-eGFP stability. Among these hits were some that could be related to ERAD: the Hsp70 chaperone HSPA13, proteasome subunits PSMB7 and PSMD13, and four subunits of the SEC61 complex [16] (preprint). Other hits, such as components of the signal recognition particle (SRP72, SRPR and SRPRB) and COQ2 cannot be directly linked to protein degradation. To control for specificity, we tested the same candidates in a control experiment using the HCMV protein US11, another HCMV protein that induces accelerated ERAD of HLA class I molecules: in this setting, no enhancement of HLA-A2-eGFP expression was observed, showing that most hits were specific to US2-mediated HLA-I degradation (Figure 1D and Figure S1).

We noted a modest, yet consistent rescue of HLA-A2-eGFP expression upon depletion of the ubiquitin-like protein UFM1 and its E1 ligase UBA5 (also known as UBE1DC1), the activating enzyme that catalyzes the first step in the UFMylation reaction (Figure 1C,E). Similar modest rescue phenotypes have been observed when other genes known to be essential, such as p97, Npl4 and Ufd1, are knocked out [16] (preprint). We introduced the UFM1- and UBA5-targeting sgRNAs in another cell type, U373 cells (Figure 1F), and confirmed that UFM1 and UBA5 have a genuine effect on HLA-A2-eGFP expression. As ubiquitination of ERAD substrates is required for recognition by the proteasome [19], the identification of two genes involved in a ubiquitination-like post-translational modification pathway suggests that UFMylation plays an important role in ERAD as well.

### 2.2. All known Players in UFMylation Affect Degradation of HLA Class I

**Figure 2:**
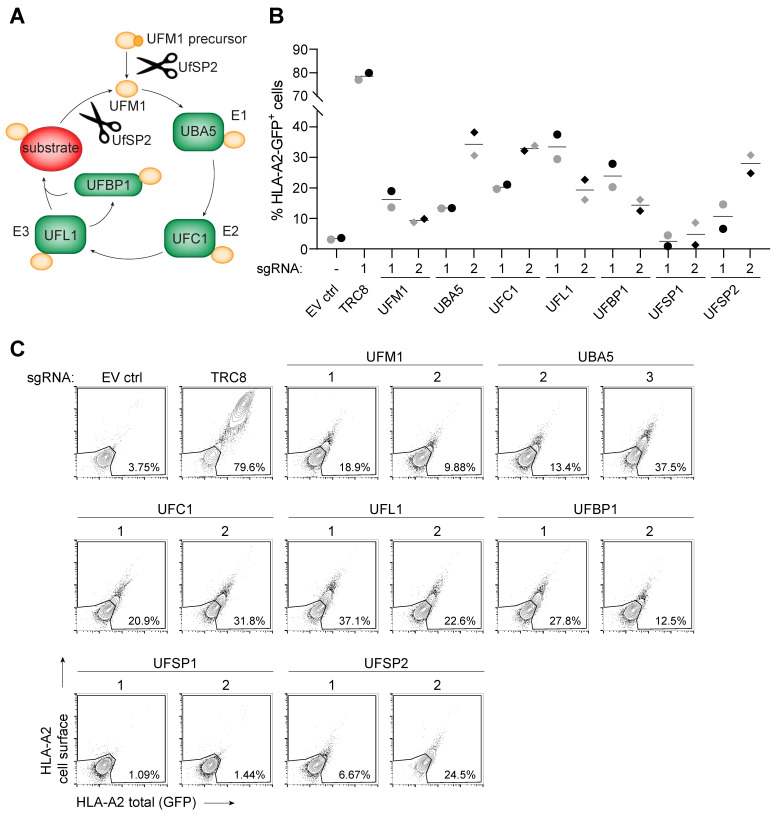
US2-mediated HLA class I expression is rescued upon knockout of multiple players in the UFMylation pathway. (**A**) Schematic overview of the UFMylation cycle. UfSP2 cleaves UFM1 downstream of its C-terminal glycine, either to create active UFM1 from its precursor, or to release UFM1 from a substrate. UFBP1 is a target of UFMylation but may also aid the UFMylation reaction. (**B**) Individual genes involved in UFMylation were targeted by two sgRNAs (circles and diamonds) and monitored for rescued HLA-A2-eGFP expression at 12 (black) or 14 (grey) days post-infection. UFSP1 was included as negative control, as this protein is only functional in murine cells but not in human tissue. The experiment was performed three times, of which one time point each of two independent experiments is shown. (The two time course experiments are shown in Figure S2). (**C**) Flow cytometry plots of the data shown in B for day 12.

As two genes from the UFM1 pathway were identified in the genome-wide CRISPR/Cas9 library screen, we tested whether other players in the UFMylation machinery also affect US2-mediated degradation of HLA-I. These factors are related to UFM1 activation and its conjugation to substrates (Figure 2A). UFM1 is initially expressed as a precursor protein that is cleaved by the UFM1-specific protease 2 (UfSP2) to yield an active UFM1. Similar to ubiquitination, UFM1 is then transferred to E1 (UBA5), E2 (UFC1) and E3 (UFL1, a.k.a. RCAD) enzymes in order to be conjugated to a substrate. UFM1-binding protein (UFBP1, a.k.a. DDRGK1) may function as a scaffold protein to aid the UFMylation reaction [20]. The cycle is completed when UfSP2 deUFMylates the substrate, freeing UFM1 for another round of UFMylation. Another deUFMylating enzyme, UfSP1, is functional in murine but not in human cells [20]. We designed two sgRNAs targeting each of these genes and introduced these in U937 cells co-expressing HLA-A2-eGFP and US2. As we observed only a minor HLA-A2 rescue effect at 14 d.p.i. (Figure 1C,E), we first tested which timepoint would be optimal to detect a phenotype upon sgRNA-targeting the UFM1 pathway (Figure S2). Targeting any of the genes involved in UFMylation resulted in reduced US2-mediated HLA-I degradation, thereby enhancing eGFP levels in a significant percentage of the cells (Figure 2B,C). As expected, only UfSP1, the inactive paralog of UfSP2, did not rescue the phenotype.

### 2.3. Clonal Knockout Cell Lines for UFM1 and UBA5 Show Stable HLA-I Rescue in the Presence of HCMV US2

**Figure 3:**
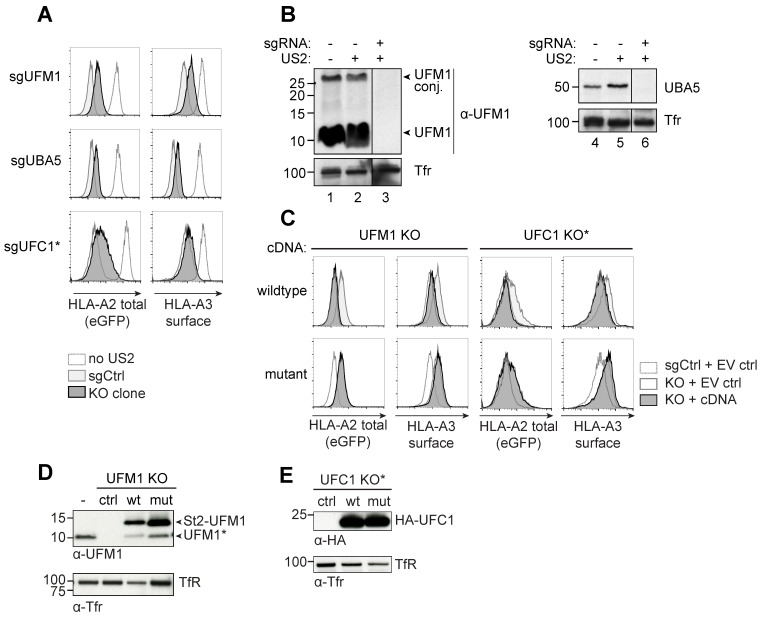
Clonal knockout cell lines for UFM1 and UBA5 show stable HLA-I rescue in the presence of HCMV US2. (**A**) U937 cells expressing HLA-A2-eGFP and US2 were transduced with sgRNAs targeting UFM1, UBA5, or UFC1. At 12 d.p.i., single HLA-A2-eGFP^+^ cells from the sgUFM1 and sgUBA5 cell lines were cloned by FACS and allowed to expand for ~8 weeks. Expression of HLA-A2-eGFP and endogenous HLA-A3 was assessed by flow cytometry. One representative clone is shown. A polyclonal cell population of UFC1-targeted U937 cells (indicated with *) expressing eGFP-tagged HLA-A2 and US2 was also stained for HLA-A3 at 10 d.p.i. These cells show a modest rescue of HLA-A2 and HLA-A3, similar to the clonal lines for UFM1 and UBA5. (**B**) Western blot analysis of the clonal cell lines established for UFM1 and UBA5. Cell lysates from the cell lines shown in A were prepared in 1% Triton X-100 and stained for the gene that was targeted by the CRISPR sgRNAs. Tfr was used as a loading control. (**C**) sgRNA-resistant wildtype, or mutant cDNAs encoding inactive UFM1 or UFC1 were transduced into the knockout clone or polyclonal cell line (for UFC1) shown in A and B. Whereas a wildtype cDNA for UFM1 reverts the HLA-A2-eGFP- and HLA-A3 rescue phenotype observed in knockout clones, the inactive mutant cDNA does not. For mutant UFM1, the four C-terminal amino acids, including glycine used for substrate conjugation, were deleted (ΔVGSC). For the inactive UFC1 cDNA, the active site cysteine essential for catalytic activity was mutated into serine (C116S). UBA5 cDNAs did not express well, therefore this cDNA was excluded from the experiment. (**D**) Immunoblots showing UFM1 protein expression in the knockout clones following the introduction of the cDNAs expressed in C. The UFM1 construct is detected using a UFM1-specific antibody. Upon expression of StrepII-tagged UFM1, a product migrating at the molecular weight of untagged UFM1 (UFM1*) is consistently observed, suggesting this product may be a truncated variant of the ST2-UFM1 construct. (**E**) Immunoblotting of the UFC1 cDNAs used in C. The HA-tagged UFC1 construct is detected using an anti-HA antibody.

To assess the phenotype of UFM1 and UBA5 knockout cells at a clonal level, we single-cell sorted HLA-A2-eGFP^+^ cells from the polyclonal knockout cultures of UFM1 and UBA5, and allowed these to establish a stable clonal population. Indeed, we were able to establish stable knockout clones for both genes and the cells displayed a moderate increase of both chimeric HLA-A2-GFP and endogenous HLA-A3 expression (Figure 3A). Protein levels of either UFM1 and UBA5 were undetectable in the respective knockout clones (Figure 3B and Figure S3). Similar, yet temporal, HLA-A2 rescue results were obtained for a polyclonal UFC1 knockout population (Figure 3A). The abrogated US2-mediated HLA-I downregulation could be fully rescued by introduction of a sgRNA-resistant cDNA vector for UFM1 (Figure 3C, top panel, and Figure 3D; note that the UFM1 cDNA encodes for the C-terminal VGSC amino acids). For UFC1, we introduced cDNAs in a polyclonal context (Figure 3C,E). The UBA5 cDNA was expressed at very low levels and no reversal of the HLA-I rescue phenotype could therefore be observed in clonal UBA5 knock-out cells (data not shown). UFM1 and UFC1 cDNAs encoding inactive mutants, lacking the 4 C-terminal amino acids (ΔVGSC) for UFM1 or harboring a C116S mutation for UFC1, could not revert the HLA-I rescue phenotype (Figure 3C, lower panels). This shows that the observed abrogation of US2-mediated HLA-I downregulation requires the activity of these proteins and is not caused by the expression of the proteins alone. To confirm that the ΔVGSC variant of UFM1, lacking the crucial glycine for conjugation to a substrate, was indeed an inactive mutant, we immunoprecipitated this construct from UFM1 KO cells (Figure S4). In ΔVGSC UFM1-expressing cells, UFMylated products are less abundant; also, this mutant was unable to bind UBA5, in contrast to wildtype UFM1 (compare lanes 6 and 7).

### 2.4. HLA Class I, US2 and p97 are not UFMylated in US2-Expressing Cells

**Figure 4:**
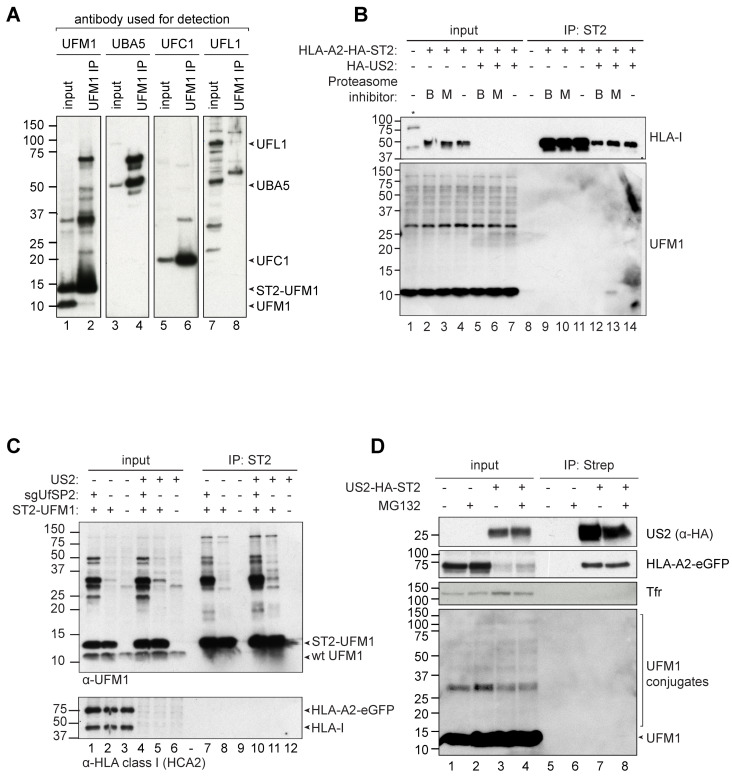
HLA class I, US2, and p97 are not UFMylated in US2-expressing cells. (**A**) ST2-UFM1 was immunoprecipitated from U937 cells co-expressing HLA-A2-eGFP and US2 in 1% LMNG lysis buffer. Samples were subsequently blotted for UFM1 to detect the interaction between UFM1 and its E1 (UBA5), E2 (UFC1) and E3 (UFL1) enzymes. Whereas UBA5 and UFL1 interact with UFM1, this could not be established for UFL1. (**B**) U937 cells expressing HLA-A2-HA-ST2 in the presence or absence of US2 were incubated with the proteasome inhibitors Bortezomib (**B**) or MG132 (M) to accumulate ubiquitinated HLA class I that would otherwise be degraded by the proteasome. HLA-A2-HA-StrepII was immunoprecipitated from these cells in 1% Digitonin lysis buffer, immunoblotted, and stained for UFMylation. (**C**) ST2-UFM1 was immunoprecipitated from U937 cells also expressing HLA-A2-eGFP, in the presence or absence of US2. For immunoprecipitation, 1% Digitonin lysis buffer was used. Indicated cell lines also contained a sgRNA targeting UfSP2. Samples were stained with a UFM1-specific antibody (**top** panel) or with HCA2, an HLA class I-specific antibody (**bottom** panel). (**D**) ST2-HA-US2 was immunoprecipitated from U937 cells also expressing eGFP-HLA-A2 in 1% Digitonin lysis buffer. Samples were immunoblotted and stained for US2, the HLA-A2-eGFP chimera (HCA2 antibody), and UFM1. Tfr was used as a loading control.

When cell lysates containing UFM1 or UFM1 immunoprecipitation samples were stained in Western blot, immunoblotting against the 9.1 kD UFM1 revealed a number of higher molecular weight proteins (Figure 4A, lanes 1 and 2), suggesting that these higher bands represent UFM1-conjugated target proteins. We performed immunoprecipitation experiments on UFM1 to assess which proteins are UFMylated in US2-expressing cells. For this, we transduced an N-terminally StrepII-tagged UFM1 (ST2-UFM1) in HLA-A2-eGFP- and US2-expressing U937 cells and pulled down UFM1 by using StrepTactin beads. Subsequent immunoblotting for UBA5 (Figure 4A, lane 4) and UFC1 (lane 6) confirmed the presence of these proteins in the UFM1 IP samples, showing that our UFM1 IP protocol was able to co-isolate UFMylated proteins from cell lysates. These UFM1-interacting factors may explain part of the higher molecular weight products observed in the UFM1 staining (Figure 4A, lane 2). We did not observe an interaction of UFM1 with the E3 enzyme UFL1 (lane 8). We also hypothesized that the ~36 kD band consistently present in all UFM1 stainings performed may represent UFBP1 (predicted weight: 35.6 kD), which was the first identified substrate of UFMylation [21]. We were however unable to detect UFBP1 in Western blot with any of the available anti-UFBP1 antibodies tested (data not shown).

We subsequently aimed at identifying US2-specific UFMylation events. As direct ubiquitination of ERAD substrates is a hallmark of proteasomal degradation, we hypothesized that UFM1 might also conjugate to HLA-I. To test this, HLA-A2-HA-StrepII was immunoprecipitated from U937 cells expressing HA-US2 or US2-lacking control cells that were pre-incubated with proteasome inhibitors MG132 or Bortezomib to accumulate ERAD-targeted HLA-I. Although ubiquitination of HLA class I was observed in US2 expressing cells upon proteasome inhibition (Figure S5 lanes 12 and 13), we did not observe UFMylation of HLA-A2-HA-StrepII (Figure 4B, lower panel). Vice versa, when StrepII-tagged UFM1 was immunoprecipitated in the presence or absence of US2, no HLA class I was detected either under the conditions used (Figure 4C). Our results suggest that HLA-I is not directly UFMylated in US2-expressing cells.

In the context of ubiquitination, the activity of de-ubiquitinating enzymes (dUbs) is a notorious obstacle for detecting targets of ubiquitination [22]. Similarly, downregulation of the deUFMylating protein UfSP2 by RNAi allows for accumulation of UFMylated target proteins [20]. Along the same lines, a CRISPR/Cas9-induced knockout of UfSP2 resulted in a strong increase of UFMylation conjugates (Figure 4C, top panel, lanes 1, 4, 7 and 10). However, UFM1-pulldown from UfSP2 knockout cells did not result in the identification of a UFMylated HLA-I species either (Figure 4C, lower panel, lanes 7 and 10).

Next, we assessed whether US2 itself could be a target of UFMylation. For this, we immunoprecipitated StrepII-HA-tagged US2 from HLA-A2-eGFP-expressing U937 cells, but could not detect UFMylated US2 molecule in the lysates by Western blot (Figure 4D). Similarly, p97, which is a target of SUMOylation [23], was not a target for UFMylation in US2 expressing cells, as assessed by immunoprecipitation on either p97 or UFM1 (data not shown). Finally, we assessed whether UFM1 and ubiquitin would influence one another. We hypothesized that, since both modifications occur on lysine residues, the two UBLs may compete with one another. Addition of the proteasome inhibitor Bortezomib or MG132, which accumulates ubiquitinated proteins, did not affect the overall UFMylation pattern or intensity (Figure 4B, lower panel, lanes 1–7). Vice versa, expression of the UfSP2 CRISPR sgRNA, which accumulates UFMylation on substrates, did not impact ubiquitination (data not shown). These findings suggest that UFM1 and ubiquitin do not compete for the same targets. Also, no ubiquitin was detected in UFM1 immunoprecipitations (data not shown), suggesting that substrates are not simultaneously modified with both UBLs.

### 2.5. Ribosomes are UFMylated in US2-Expressing Cells

**Figure 5:**
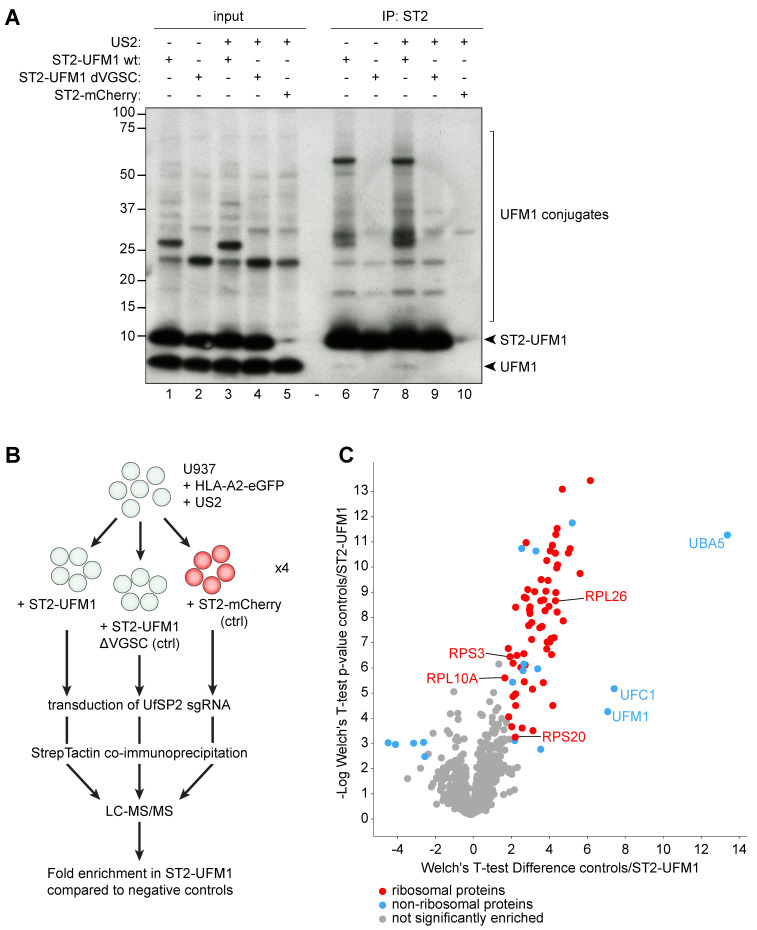
Mass spectrometry identifies ribosomal UFMylation in the presence of US2. (**A**) U937 cells expressing HLA-A2-eGFP in the presence of absence of US2 were transduced with StrepII-tagged UFM1 wildtype, a StrepII-tagged ΔVGSC mutant of UFM1, or StrepII-tagged mCherry. A sgRNA targeting UfSP2 was subsequently introduced in all cell lines. StrepTactin co-immunoprecipitations were performed in 1% LMNG to pull down UFM1 or control substrates. Immunoblotting was performed with an antibody staining against UFM1. (**B)** Schematic set-up of the cell lines and workflow used for mass spectrometry. (**C**) Volcano plot showing the proteins identified by mass spectrometry upon StrepII-UFM1 immunoprecipitation. The enrichment per protein was calculated compared to the negative control cell lines. Significantly enriched (**top** right) or depleted (**left**) hits are shown in color, while the gray dots show proteins that are not significantly enriched. Red hits represent ribosomal proteins, while all other hits are shown in blue. A complete list of names and functions of the proteins that were significantly enriched or depleted is presented in Supplementary information 2.

As we did not detect UFMylation of HLA-I, US2, nor p97 in US2 expressing cells, we turned to mass spectrometry to identify UFMylated proteins. To this end, we transduced US2-expressing HLA-A2-eGFP U937 cells with either StrepII-UFM1, an inactive UFM1 mutant (StrepII-UFM1 ΔVGSC), or StrepII-mCherry, followed by introduction of the UfSP2-targeting sgRNA in all cell lines. Upon immunoprecipitation of the three StrepII-tagged proteins, we analyzed the distribution of UFM1-conjugates by Western blot (Figure 5A). As expected, we observed increased levels of UFMylated proteins in StrepII-UFM1 cells as compared to control StrepII-UFM1 ΔVGSC and StrepII-mCherry cells. This difference was apparent in both the absence (Figure 5A, compare lane 7 to lane 6) and presence (lanes 9 and 10 versus lane 8) of US2.

For mass spectrometry, quadruplicate immunoprecipitations were performed on the sgUfSP2-expressing UFM1- and control cell lines. These were subsequently subjected to mass spectrometry (Figure 5B). The enrichment of co-precipitated proteins in the UFM1 sample was compared to the negative control cell lines (Figure 5C; significantly enriched proteins determined upon two-sided Welch’s t-test, permutation-based FDR = 0.01, S0 = 1, *n* = 4). As expected, the strongest hits were directly involved in the UFMylation pathway: UFM1, UBA5 and UFC1, confirming the clear interactions we observed previously (Figure 4A). Additionally, multiple significantly enriched proteins were detected in the StrepII-UFM1 expressing cells (such as UPF1, GNB2L1 and TXNL1) that were not previously linked to ERAD (Supplementary information 2). A large number of ribosomal proteins were identified (Figure 5C, red dots), suggesting that multiple ribosomal subunits are either directly UFMylated, or interact with other UFMylated proteins. Four ribosomal proteins, uS3 (RPS3), uS10 (RPS20), uL16 (RPL10), and RPL26 have previously been described to be UFMylated [24,25] and are also observed among the ribosomal proteins enriched in our experiment (Figure 5C and Supplementary information 2). Additional proteins found to be enriched (e.g., EIF1AX, FAU and TP53RK), though not structural components of the ribosome (blue datapoints in Figure 5C), are nonetheless related to ribosome function or translation (Supplementary information 2).

### 2.6. ER-to-Cytosol Dislocation of HLA Class I is Reduced in UFM1 Knockout Cells

**Figure 6:**
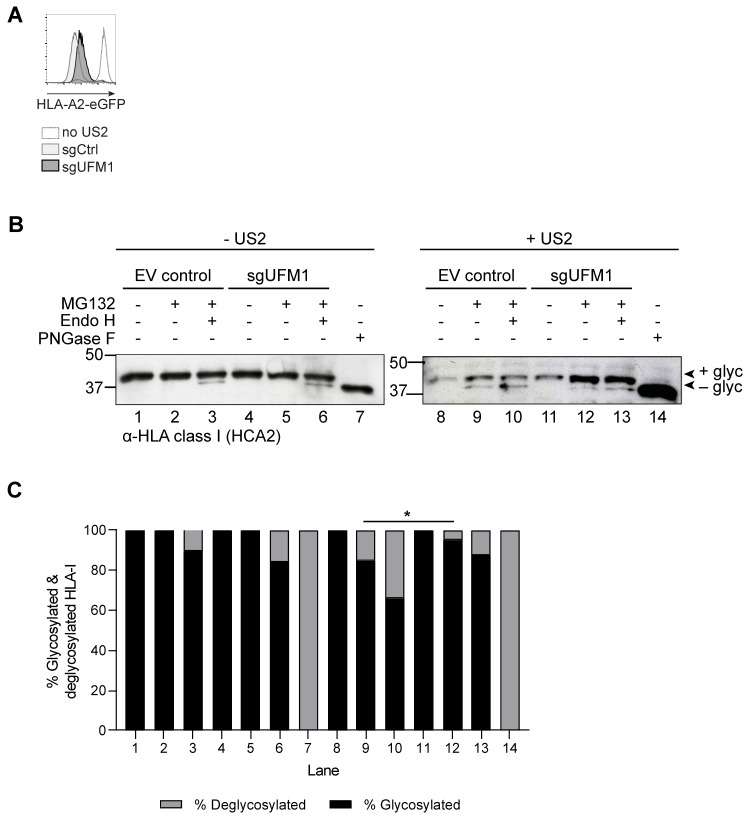
Functional UFM1 is required for efficient HLA-I dislocation to the cytosol. (**A**) U373 cells stably expressing HLA-A2-eGFP, US2, and Cas9 were transduced either with sgUFM1 or with an empty vector control (sgCtrl). HLA-A2-eGFP expression of the polyclonal cell population was assessed by flow cytometry at 14 days post-transduction. (**B**) The U373 cells shown in A were treated overnight with the proteasome inhibitor MG132 or DMSO control, after which they were lysed for western blot analysis. The cell lysates were treated with EndoH, PNGaseF, or control as indicated. This experiment was performed three times, of which one representative blot is shown. (**C**) Quantification of (**B**). The change in proportions of glycosylated/non-glycosylated HLA-I upon introduction of sgUFM1 (lane 9 versus 12) is significant (Fisher’s exact test, * *p* < 0.05).

Next, we assessed whether ER-to-cytosol transport (dislocation or retro-translocation), is dependent on UFM1. To this end, we used HLA-A2-eGFP-expressing U373 cells with or without US2, transduced to express a sgRNA targeting UFM1 or a control. In the sgUFM1-expressing U373 cells, HLA-A2 is rescued to a similar extent as in U937 cells (Figure 6A and Figure 3A, respectively). The U373 cells were used instead of U937 because of their higher survival rate upon treatment with proteasome inhibitor.

In the presence of US2, HLA-I is deglycosylated by a cytosolic *N*-glycanase upon exit from the ER [15,26]. Degradation of the cytosolic, deglycosylated degradation intermediate can be prevented by treating the cells with proteasome inhibitor. Due to the involvement of N-glycanase, cytosolic HLA-I can be distinguished from ER-resident HLA-I on the basis of its glycosylation status and concomitant migration in SDS-PAGE. Overnight treatment of US2-expressing U373 cells with a low concentration of the proteasome inhibitor MG132 allows detection of dislocated, deglycosylated HLA-I (Figure 6B, compare lanes 8 and 9). In cells not expressing US2, no deglycosylated HLA-I was visible upon treatment with proteasome inhibitor (compare lanes 1 and 2). Endoglycosidase H (EndoH)-treatment was used to obtain more information on the composition of the N-linked glycans of HLA-I molecules and, hence, their location in the cell. EndoH can only cleave immature N-linked glycans present on ER-resident glycoproteins, but not mature glycans present on proteins that have migrated to the Golgi and beyond. EndoH treatment of samples from cells not expressing US2 results in only a small proportion of deglycosylated HLA-I (compare lanes 2 and 3), indicating that the majority of MHC-I has exited the ER. Treatment of samples with peptide N-glycanase-F (PNGase F), that cleaves all glycans regardless of their maturation status, was included to visualize the mobility of deglycosylated HLA-I (lanes 7 and 14). In US2-expressing UFM1 KO cells, rescue of HLA-I can be observed compared to control cells (Figure 6B, compare intensity of the band in lane 11 vs 8). The HLA-I in the sgUFM1 cells migrates at the level of intact, glycosylated HLA-I, suggesting that HLA-I is no longer dislocated to the cytosol in the absence of functional UFM1. This finding suggests that UFM1 acts at the level of ER-to-cytosol dislocation of the substrate. Upon treatment with proteasome inhibitor, glycosylated, intact HLA-I remains the dominant species in UFM1 KO cells (lane 12). In addition, the fraction of deglycosylated HLA-I is decreased in US2-expressing UFM1 KO cells (Figure 6B,C, lane 12 vs 9), also pointing towards a role of the UFMylation pathway in influencing the rate of dislocation of HLA-I. This suggests that dislocation of HLA-I is compromised in the UFM1 KO cells. Digestion of these samples with EndoH does not change the migration pattern (lane 13). As EndoH only cleaves glycans on ER-resident proteins, but not on those migrated downstream of the ER, the EndoH-resistance of the majority of HLA-I molecules in the UFM1 KO cells suggests that these proteins have likely exited the ER and moved further down the secretory pathway. The latter may explain the increased levels of HLA-I at the surface of UFM1 KO cells (Figure 2C and Figure 3A,C). The exact localization and traficking of HLA-1 in cells with modifications of the UFM1 pathway awaits further investigation.

Taken together, our findings suggest that UFMylation contributes ER-to-cytosol dislocation of HLA class I, as a part of US2-induced ER-associated degradation of HLA-I molecules.

## 3. Discussion

Here, we describe a genome-wide CRISPR/Cas9 screen to identify cellular factors involved in HCMV US2-mediated ERAD of HLA-I. We identified multiple genes that were previously linked to US2-mediated HLA-I downregulation, including the ubiquitin ligase TRC8, the E2 enzyme UBE2G2, and p97, the ATPase facilitating dislocation of ERAD substrates. Some genes, such as UBXD8 (FAF2) and the p97 co-factors Npl4 and Ufd1, have previously been described in ERAD, but did not affect US2 function in those studies [14,18]. Although we have not validated these hits in detail, targeting them with CRISPR sgRNAs results in abrogation of US2-mediated HLA class I downregulation [16] (unpublished). The discrepancy with previous studies may arise from the different techniques used: in contrast to our knockout approach, Npl4 and Ufd1 were previously knocked down using siRNAs [14], which does not result in full target knockdown, while UBXD8 was studied in a pulse-chase approach to assess HLA class I dislocation [18].

We show that knockout of genes involved in the UFM1 pathway moderately, yet consistently, hamper US2-mediated HLA-I degradation. A link between UFM1 and the ER has previously been described [27], yet a role in protein degradation has been reported only recently [25,28]. UFM1 is a post-translational modifier structurally related to ubiquitin [29]. Similar to ubiquitin, UFM1 is conjugated to its substrates via an iso-peptide bond between the C-terminal glycine of UFM1 and a lysine residue of the substrate [30]. For both modifiers, the conjugation to substrates is facilitated by E1, E2 and E3 enzymes. However, UFM1 does not seem to function in ERAD the same way as ubiquitin: while the degradation substrate HLA-I becomes ubiquitinated, UFMylation of HLA-I could not be detected. Similarly, UFMylation did not take place on US2 or proteins directly related to US2-mediated ERAD. Therefore, the mechanism behind UFM1′s impact on protein degradation remains to be clarified. This, in combination with the subtle HLA-I rescue phenotypes observed upon knocking out players of the UFMylation pathway, suggests that UFM1 may play an indirect role in protein degradation, potentially by affecting the protein sorting mechanisms of the cell.

The results from our genome-wide CRISPR/Cas9 library screen are in agreement with screens that have been performed using different model systems. One of these recently described genome-wide CRISPR/Cas9 library screens has identified the UFM1 pathway to regulate SQSTM1 expression in an ER-stress-dependent manner [31]. Another screen, aimed to identify genes involved in ERAD, identified a similarly modest role for UFM1 [25]. Several additional studies have attempted to identify targets for UFMylation, mostly by mass spectrometry-based approaches. Despite these efforts, only few UFM1 substrates have been identified to date. The first-identified UFM1 target [21], UFBP1, was later suggested to play a role in the UFMylation pathway itself [20,27,32]. Other targets include LZAP, a binding partner of UFL1, the ribosome [24,25], and, interestingly, multiple chaperones of the Hsp40 and -70 families, such as DNAJC1, HSPA8, and BiP [20,32]. While our genome-wide screen also identifies a Hsp70 (HSPA13) and Hsp40 chaperone (SEC63), they did not associate with UFM1 in our mass spectrometry analysis. Although UFM1 is ubiquitously expressed in many tissues [21], its target proteins may differ between cell types and the experimental context used. In B cells in particular, UFMylation of UFBP1 has been shown to act upon different branches of the unfolded protein response (UPR) to promote the differentiation and function of plasma cells [33].

We identified many ribosome subunits as potential targets for UFMylation in US2-expressing cells. UFMylation of RPS3, RPS20 and RPL10 [24], and of RPL26 [25,28] has been described previously, suggesting that these ribosomal proteins are genuine targets of UFMylation. Because RPS3, RPS20 and RPL10 are located near the mRNA entry channel in the large ribosomal subunit, it has been suggested that UFMylation may affect mRNA entry into the ribosome [24]. RPL26 on the other hand is located closely to the polypeptide exit tunnel. This, in combination with the finding that UFMylated RPL26 interacts with the SEC61 complex, suggests that UFMylation of the ribosome may regulate translation and translocation efficiency, although no direct effect on protein translation could be observed [25,28]. The changes US2 promotes in the ER upon directing HLA class I for degradation might consequently affect protein sorting indirectly, in a UFM1-dependent manner. As UFMylation of the ribosome may affect translation efficiency [24,25,28], and US2 may be particularly sensitive to changes in translational efficiency as a consequence of its inefficient signal peptide [16] (preprint), the rescue phenotype could alternatively arise from altered US2 expression. Due to the low expression levels of US2 and the lack of a suitable detection antibody, we were unable to assess this option. Finally we cannot exclude that changes in the UFMylation pathway may alter the translation efficiency of HLA-I itself, and of ERAD-related proteins.

In the same screen that identified RPS3, RPS20 and RPL10, UFMylation of eIF6 was observed [24]; eIF6 is a translation initiation factor that prevents association between the 40S and 60S ribosomal subunits. We identified eIF1AX, another translation initiation factor associated with the 40S ribosome. None of the proteins found to interact with UFM1 in our mass spectrometry studies are functionally related to ER-associated degradation, and it was recently suggested that the identification of UFM1 in the context of ERAD may be an indirect consequence of UFMylation being related to maintaining ER homeostasis [25], which may be perturbed when inducing protein degradation. Upon chemically-induced ER stress, UFM1 is upregulated via the transcription factor Xbp1s [34], and UFMylation allows cells to survive ER stress by suppressing apoptosis [20,27,32,35]. More specifically, UFBP1 (also known as DDRGK1 or C20orf116) is an ER membrane protein that binds the ER stress protein IRE1α in an UFM1-dependent manner [27]. IRE1α in turn cleaves Xbp1, that functions as a transcription factor to activate ER chaperones as well as UFM1 [34]. A positive feedback loop may arise during ER stress, as the elevated expression of UFM1 potentially stabilizes additional IRE1α molecules.

The UFMylation pathway could be related to ER stress via UFBP1. In the absence of UFM1, IRE1α is a substrate for SEL1/HRD1-mediated ERAD. IRE1α is not UFMylated itself, but it is rescued from degradation by binding to UFMylated UFBP1. Hence, depending on the UFMylation status of UFBP1, the protein can rescue ERAD substrates from degradation. Also the ER chaperone BiP has been shown to be an indirect target of UFMylation by interacting with UFBP1 [32]. UFBP1 may thus act as a regulator of protein stability, depending on its UFMylation status. By switching the UFMylation status of UFBP1, a far larger number of targets may be regulated via protein-protein interactions without the need for direct UFMylation of these target proteins themselves. In fact, it has been recently shown that UFBP1/DDRGK1 brings the E3 ligase UFL1 to the ER surface, where RPN1 and RPL26 are UFMylated. This UFMylation at the ER seems to facilitate ER-phagy in conditions of starvation and to repress the unfolded protein response [36].

In addition to the underlying mechanism, the substrate specificity of the UFM1 pathway remains to be established. In our unbiased CRISPR/Cas9 screen, all known factors of the UFMylation pathway were found to affect HLA-I degradation via US2, but not US11, suggesting substrate specificity. The contribution of the UFM1 pathway to degradation of other cellular proteins remains to be established.

US2-mediated HLA-I downregulation at the cell surface is partly restored upon knockout of genes involved in the UFMylation pathway. Also total levels of HLA-I increase within the cell upon inhibition of the UFM1 pathway. In the absence of UFMylation, US2-dependent ER-to-cytosol dislocation appears to be compromised. However, nor HLA class I, nor US2, nor proteins previously shown to be involved in US2-mediated ERAD, were found to be UFMylated. The impact of the UFM1 pathway on HLA-I degradation may, therefore, be indirect: UFMylation is known to play an important role in maintaining ER homeostasis, and US2-mediated protein degradation likely acts as a disturbing factor. The ins-and-outs of this dysregulation require further investigation.

## 4. Materials and Methods

### 4.1. Cell Culture and Lentiviral Transduction

U937 cells (ATCC) were cultured in RPMI 1640 culture medium (Gibco) supplemented with penicillin/streptomycin (Gibco), Ultraglutamine-1 (Gibco) and 10% fetal calf serum (BioWest). Wildtype U937 cells were lentivirally transduced with HLA-A2-eGFP (kindly provided by Dr. Louise Boyle, University of Cambridge UK) expressed from a lentiviral pSicoR vector containing a hygromycin B resistance gene. Successfully transduced cells were selected with Hygromycin B at 3 days post-infection, and subcloned. The HLA-A2-eGFP cells were subsequently transduced with US2 under control of an EF1a promoter (pSicoR-EF1A-US2 (RP-549)) and a clonal cell line was established by fluorescence-activated cell sorting (FACS) of the HLA-A2-eGFPlow cells. Knock-out (KO) of genes involved in the UFMylation pathway was achieved by CRISPR/Cas9 guided genome editing (see below for details). Rescue of protein expression in KO cells was achieved by expression of sgRNA-resistant cDNAs from the BIC-PGK-Zeo vector.

U373 cells (ATCC) were cultured in Dulbecco’s Modified Eagle Medium (DMEM; Gibco) supplemented with penicillin/streptomycin (Gibco), Ultraglutamine-1 (Gibco) and 10% fetal calf serum (BioWest). U373 cells were transduced with the abovementioned HLA-A2-eGFP vector and selected in the same manner (Hygromycin B at 3 days post-infection). A polyclonal cell line was established after fluorescence-activated cell sorting (FACS) of the HLA-A2-eGFP+ cells. Cells were subsequently transduced with an HA-tagged US2 and selected with Blasticidin at day 3 post-infection. CRISPR/Cas9 genome editing was performed as described below.

### 4.2. Plasmids

HLA-A2-eGFP (kindly provided by Dr. Louise Boyle, University of Cambridge UK) was subcloned in a bidirectional lentiviral expression vector derived from no.2025.pCCLsin.PPT.pA.CTE.4 × -scrT.eGFP.mCMV.hPGK.NG-FR.pre (kindly provided by L. Naldini, San Raffaele Scientific Institute, Milan, Italy) as described elsewhere [10]. This lentiviral vector (BIC HLA-A2-EGFP_HygB-T2A-mCD4 (RP-31)) contains a human EF1A promoter to facilitate potent expression of HLA-A2-eGFP and expresses an Hygromycin B resistance gene fused with a T2A sequence to a tail-less mouse CD4 under control of a different promoter (PGK).

US2 was expressed from several backbone vectors. Untagged US2 was expressed in U937 cells from the pSicoR-EF1A-US2 vector (RP-549)). This vector was constructed from the pSicoR vector [37], from which the U6 promoter was removed and the CMV promoter was replaced by an EF1A promotor. US2leader-HA-US2 was expressed in U373 cells from BIC-PGK-Blast (RP138), derived from no.2025.pCCLsin.PPT.pA.CTE.4 × -scrT.eGFP.mCMV.hPGK.NG-FR.pre. This lentiviral vector contains a human EF1A promoter to facilitate potent expression of the downstream cloned gene and expresses the BlastR selection marker from a different promoter (PGK).

For CRISPR/Cas9 genome engineering, single guide RNAs (sgRNAs) were introduced in the lentiviral pSicoR-CRISPR-PuroR vector (RP-557) [38] as described previously. To allow for rescue of protein expression in KO cells, we cloned cDNAs, UFM1 and UFC1 inactive mutant controls in dual promoter lentiviral vectors (BIC-PGK-Zeo (RP137), which is the same vector as BIC-PGK-Blast (RP138), yet with ZeoR selection instead of BlastR. We introduced silent mutations in the sgRNA target sites to prevent CRISPR/Cas9 mediated editing of lentiviral cDNA sequences in CRISPR/Cas9-harbouring KO cells.

### 4.3. Genome-Wide CRISPR/Cas9 Library Screen

150 million U937 HLA-A2-eGFP cells co-expressing US2 and SpCas9 were transduced at an MOI of 2 in duplicate with the human GeCKOv2 CRISPR knockout pooled library (obtained from Feng Zhang (Addgene, Watertown, NY, USA). The library targets 19,050 genes with 6 sgRNAs/gene. Transduced cells were selected by puromycin treatment (2 μg/mL) at 2 days post-infection (d.p.i.) and maintained at high complexity for the duration of the screen. At 7 and 18 d.p.i., 1 billion cells were harvested and subjected to cell sorting via a two-step sort-protocol using a Becton Dickinson Influx cell sorter. First, PE^+^ cells were sorted using an ‘enrichment-protocol’, which allowed for high-speed cell sorting of the entire population of cells in a short timeframe. Next, we sorted the top ±1% of eGFP^+^/PE^+^ cells to purity selecting for cells that display enhanced levels of eGFP and HLA-A2 surface staining (stained with BB7.2-PE; BD Biosciences, #558570). As control, the eGFPlow/HLA-A2low were sorted. We next isolated genomic DNA from all sorted cell populations by standard phenol/chloroform extraction protocols using the Phase Lock gel heavy tubes (Quantabio; 10847-802) according to manufacturer’s instructions. Next, the lentiviral sgRNA inserts were PCR-amplified for 27 cycles using Fw primer 5′-**AATGATACGGCGACCACCGAGATCTACAC**TCTTTCCCTACACGACGCTCTTCCGATCTNNNNNNcttgtggaaaggacgaaacacc-3′ and Rev primer 5′-**CAAGCAGAAGACGGCATACGAGAT**gactcggtgccactttttcaag-3′ and Phusion polymerase (NEB) in the presence of buffer GC supplemented with DMSO. Both primers contain an Illumina adapter sequence (displayed in bold) that allows for direct loading on an Illumina NextSeq500 sequencer and a lentiviral-specific primer binding site (lowercase letter) to facilitate amplification of the integrated lentiviral sgRNA sequence. The Fw primer also contains a unique 6nt barcode sequence (NNNNNN) allowing for multiplexed sequencing, and a primer binding site for the Illumina sequencing primer (underlined). The PCR products were purified/concentrated using a PCR purification kit (Qiagen, Hilden, DE), and subsequently loaded on a 20% polyacrylamide gel in 0.5 × TBE. Bands of the correct size were excised, electro-eluted, purified by phenol-chloroform extraction and subsequently quantified using a Nanodrop quantification device (Nanodrop, Rockland, DE, USA) and an Agilent bioanalyzer (Agilent Technologies, Palo Alto, CA, USA). Deep sequencing was carried out as single 75bp run on a Illumina NextSeq500 machine (performed by the Utrecht Sequencing facility USEQ) using the sequencing primer 5′-ACACTCTTTCCCTACACGACGCTCTTCCGATCT-5′, in which the Index and sgRNA sequence was sequenced simultaneously. Due to the low complexity at the start of the sequence, a Phix library was mixed with the libraries to 20% of total reads. Sequences were aligned to the sgRNA library by using Bowtie2 [39] and the counts per sgRNA were calculated. We used the MaGeCk package [40] (available from https://sourceforge.net/projects/mageck/) as a computational tool to identify genes that were significantly enriched in the screens by comparing sgRNA read counts of control sorted cells to cells displaying enhanced HLA-A2-eGFP levels. The overlap between the top 300 of the two duplicates was compared and used to select genes for further validation. The hits in this list were ranked, based on the number of sgRNAs that was enriched >5-fold, and the number of sgRNAs that showed >20-fold enrichment. As every gene was targeted with 6 sgRNAs in duplicate, the genes that showed >5-fold enrichment in at least 6 out of the total 12 sgRNAs (from both duplicates) were selected for further validation. In total, this list contained 46 genes. For initial validation studies, two sgRNAs/gene from the GeCKOv2human library that yielded the highest enrichment were selected and cloned into a pSicoR lentiviral vector with an EFS-PuroR-T2A-Cas9 cassette. sgRNA sequences are listed in Supplementary information 1. sgRNAs were transduced in target cells, and transduced cells were selected for by puromycin selection (2 μg/mL). HLA-A2-eGFP rescue of these hits was validated based on eGFP intensity and an HLA-A2-specific antibody staining on the cell surface, using a flow cytometric readout (BD FACS Canto II). When setting up the validation, some gene-knockouts resulted in strong autofluorescence, indicating that these were false-positive hits. We therefore included the irrelevant PE-Texas Red channel to omit PE-Texas Red^+^ cells prior to assessing HLA-A2-eGFP rescue. HLA-A2-eGFP expression was measured using the FITC channel for eGFP and the PE channel for HLA-A2 cell surface expression (using a PE-conjugated HLA-A2-specific antibody, BB7.2, see Section 4.5). Genes that showed only autofluorescent signal were omitted from further analysis and are not shown in the list from Figure 1B. Validation was performed on day 7, 11, 14, 18 and 28 post infection. Target site sequences of these additional sgRNAs are also listed in Supplementary information 1.

### 4.4. Clonal Knockout Cell Lines

sgRNAs targeting UFM1-, UBA5-, and UFC1 were introduced in target cell lines by lentiviral transduction. At 3 days post-infection, transduced cells were selected for by Puromycin treatment (2 μg/mL). At 10 d.p.i., the sgUFM1 and sgUBA5 cells were stained for HLA-A2 cell surface expression and PE^+^/GFP^+^ cells were single-cell sorted by fluorescence-activated cell sorting (FACS) on a FACSAria III. Cells were allowed to recover for ~8 weeks and analyzed by flow cytometry to select cells that displayed enhanced HLA-A2-eGFP and endogenous HLA-A3 expression. The knock-out status was confirmed by Western blot and the genomic target sites of both alleles were sequenced by Sanger sequencing.

### 4.5. Antibodies

Antibodies used in this study were: rabbit anti-UFM1 [EPR4264(2)] (Abcam, ab109305), rabbit anti-UBA5 (Abcam, ab177507), rabbit anti-UFC1 [EPR15014-102] (Abcam, Cambridge, UK, AB189252), rabbit anti-UFL1 (Atlas Antibodies, Bromma, Sweden, HPA030559), mouse anti-human transferrin receptor (H68.4) (Invitrogen, Carlsbad, CA, USA, 13-6800), mouse anti-p97 (VCP) (BD Transduction Laboratories, San Jose, CA, USA, 612183), rat anti-HA (3F10) (Roche, Woerden, NL, 11867423001), mouse anti-HLA class I HCA2, mouse anti-HLA class I HC10, mouse anti-ubiquitin (P4D1) (Santa Cruz, Dallas, TX, USA, sc-8017), goat anti-rabbit-HRP (light chain-specific) (Jackson Immunoresearch, Ely, UK, 211-032-171), goat anti-rat-HRP (light chain-specific) (Jackson Immunoresearch, Ely, UK, 112-035-175), goat anti-mouse-HRP (light chain-specific (Jackson Immunoresearch, Ely, UK, 115-035-174), mouse anti-HLA-A2-PE (BB7.2) (BD Biosciences, San Jose, CA, USA, 558570), human anti-HLA-A3 (OK2F3), goat anti-human-PE (Jackson Immunoresearch, Ely, UK, 109-116-127).

### 4.6. Immunoblotting

When indicated, cells were incubated overnight with 500 nM MG132 (Sigma-Aldrich, Zwijndrecht, NL, C2211-5MG), 5 nM Bortezomib (New England Biolabs, Ipswich, MA, USA, 2204S) or DMSO control, prior to preparing cell lysates. To make lysates, cells were counted using a Casy cell counter and an equal number of live cells was subjected to two washes in PBS containing 20 mM N-ethylmaleimide (Sigma-Aldrich, Zwijndrecht, The Netherlands, E3876-5G) to block de-ubiquitinating and de-UFMylating activity. Subsequently, cells were lysed on ice in Triton X-100 lysis buffer (1% Triton X-100, Applichem Panreac, Darmstadt, DE, A13880500) 100 mM NaCl, 50 mM Tris, pH 7.5) supplemented with 20 mM N-ethylmaleimide. Samples were spun down at 12,000× *g* for 20 min at 4 °C to pellet cell debris and nuclei. Supernatant was transferred to a clean tube and mixed with Laemmli sample buffer containing DTT. Lysates were stored at −80 °C until further use. For Western blot analysis, proteins were separated using SDS-PAGE (Thermo Bolt 4-12% or self-made gels) and subsequently transferred to PVDF membranes (Merck Millipore, Darmstadt DE, IPVH00010). Membranes were blocked using 5% milk and incubated with the respective antibodies for specific protein detection. Protein bands were visualized using ECL (Thermo Scientific Pierce, Landsmeer, The Netherlands) on Amersham Hyperfilm ECL films (GE Healthcare, Buckinghamshire, UK). Samples subjected to either EndoH or PNGaseF treatment were incubated at 37oC for 1 h with EndoH (New England Biolabs, Ipswich, MA, USA, P0702L) or GlycosidaseF (Roche, Woerden, The Netherlands), respectively, in the appropriate glycobuffer. Quantification of total and deglycosylated HLA-I was performed using ImageJ software (National Institutes of Health, Bethesda, MD, USA).

### 4.7. Co-immunoprecipitation

Cells were lysed in 1% Digitonin (Calbiochem, Darmstadst, DE) lysis buffer (pH 7.5) containing 50 mM Tris-HCl, 5 mM MgCl2 and 150 mM NaCl, supplemented with 1 mM Pefabloc SC (Roche), 10 μM Leupeptin (Roche) and 20 mM *N*-ethylmaleimide (Sigma-Aldrich, Zwijndrecht, NL). Lysates were incubated on ice for 60 min and subsequently centrifuged at 12,000× *g* for 20 min at 4 °C to remove nuclei and cell debris. Post-nuclear lysates were incubated overnight with StrepTactin beads (GE Healthcare, Buckinghamshire, UK,). Beads were washed 4 times with 0.1% Digitonin lysis buffer, after which they were eluted for 45 min on ice. Elution buffer contained 2.5 mM d-Desthiobiotin, 150 mM NaCl, 100 mM Tris-HCl and 1 mM EDTA, at a pH of 8.0. The eluate was collected from the beads using SpinX columns (Corning Costar, Amsterdam, The Netherlands) and was denatured in Laemmli sample buffer containing DTT. Immunoblotting was performed as described before.

### 4.8. Mass Spectrometry

U937 cells containing HLA-A2-eGFP and US2 were transduced with either StrepII-tagged UFM1 wildtype cDNA or one of two control constructs: StrepII-tagged UFM1 ΔVGSC (inactive mutant lacking the 4 C-terminal residues) or StrepII-tagged mCherry. Per cell line, 50 million cells were pelleted in quadruplicate. Cell pellets were stored at −80 °C until lysis and immunoprecipitation. For affinity enrichment, pellets were lysed on ice using TAP lysis buffer (50 mM Tris pH 7.5, 100 mM NaCl, 5% (*v*/*v*) glycerol, 0.2 % (*v*/*v*) Nonidet-P40, 1.5 mM MgCl2, 1µg/mL Avidin (2-0204-015; IBA) and protease inhibitor cocktail (EDTA-free, cOmplete; Roche, Woerden, NL) for 15 min followed by a 5 min sonication step at 4 °C. StrepTactin agarose was added to the clarified cell lysates and incubated for 3 h at 4 °C on a rotary wheel. Beads were washed four times in TAP lysis buffer to reduce the concentration of unspecific proteins and to separate specific binders from background ones. Samples were washed five additional times with TAP washing buffer (50 mM Tris pH 7.5, 100 mM NaCl, 5% (*v*/*v*) glycerol, 1.5 mM MgCl2), to remove remaining detergents. Beads were re-suspended in 20 µl guanidinium chloride buffer (6 M GdmCl, 10 mM TCEP, 40 mM CAA, 100 mM Tris/HCl pH 8), boiled at 95 °C for 5 min and digested by adding 20 µl LysC-Protease-Mix (100 mM Tris/HCl pH 8 and 0.5 µg LysC) for 3 h at 30 °C. Samples were diluted 1:5 with Trypsin-Protease-Mix (100 mM Tris/HCl pH 8 and 1 µg Trypsin) (Promega, Madison, WI, USA) and incubated for 12h at room temperature. TFA and acetonitrile was added to a final concentration of 0.6% and 2%, respectively. Peptides were desalted and concentrated using C18 Empore filter discs (3M, Eagan, MN, USA). After elution, peptides were analysed employing an EASY-nanoLC1200 system (Thermo Fisher Scientific, Leiden, NL), which was directly coupled to a Q-Exactive plus HF mass spectrometer (Thermo Fisher Scientific, Leiden, The Netherlands). Peptides were loaded on an analytical 50 cm C18 column (50 cm, 75 µm column diameter; ReproSil-Pur C18-AQ 1.9 µM resin; Dr. Maisch, Ammerbuch-Entringen, DE) and eluted using an 120 min acetonitrile gradient starting with 5% to 30% (95 min), 30% to 60% (5 min), 60% to 95% (5 min), a wash out period of 5 min at 95%, 95% to 5% (5 min) and a re-adjustment phase at 5% of organic acetonitrile buffer (80% acetonitrile, 0.1% Formic acid) (5 min) at a constant flow of 300 nL/min. The mass spectrometer was used in a data-dependent acquisition mode with one full MS scan, followed by 15 MS/MS scans. Raw files were processed with MaxQuant version 1.5.3.34 (Max-Planck Institut, Martinsried, DE) using label-free quantification (LFQ) and match between run options and searched against forward and reverse sequences of the human proteome (UniprotKB, release 03.2016, The UniProt Consortium, https://www.uniprot.org) by the built-in Andromeda search engine. Carbamidometylation was set as fixed, methionine oxidation and *N*-acetylation as variable modification. Peptide and protein identification were controlled by a False Discovery Rate (FDR) of 0.01. Perseus version 1.5.3.0 (Max-Planck Institut, Martinsried, DE) was used to analyze the output of MaxQuant. Protein groups identified as known contaminants or reverse sequence matches were excluded from the analysis. Only proteins with a minimum of 2 LFQ quantifications in at least one group of replicate experiments (N = 4) for a specific condition were considered for the analysis. Missing values were imputed using normal distribution, whose standard deviation was defined as 30% and the mean was offset by −1.8 standard deviations of the data distribution of the real intensities observed in the corresponding MS run, respectively. The significance of the protein enrichment in the pulldowns of a bait *versus* the other condition was determined by Welch’s t-test (two-sided, S0 = 1) and corrected for multiple hypothesis testing using permutation-based false discovery rate statistics (FDR = 0.01, 250 permutations).

## Data Availability

The data presented in this study are available in insert article and Supplementary Material.

## Supplementary Materials

The following are available online, Figure S1: Validation of the genome-wide library screen at 7, 11, 18, and 28 days post-infection. Figure S2: Determination of the optimal timepoint for detecting HLA-I rescue. Figure S3: Clonal knockout cell lines for UFM1 and UBA5 show stable HLA-I rescue in the presence of HCMV US2. Figure S4: Mutant UFM1 lacking its C-terminus is unable to bind UBA5. Figure S5: HLA class I is ubiquitinated in US2 expressing cells upon proteasome inhibition. Supplementary information 1: Target genes and genomic target sites of sgRNAs used in this study. Supplementary information 2: List of significantly enriched/depleted UFM1-binding proteins.

## Sample Availability

Samples of the compounds used in this study are available from the authors.
