# Supplementary material for: The UFM1 Pathway Impacts HCMV US2-Mediated Degradation of HLA Class I
Source: Molecules. 2021 Jan 8;26(2):287. doi: 10.3390/molecules26020287 (PMC7827699; doi:10.3390/molecules26020287)

Figure 1: A genome-wide CRISPR/Cas9 screen identifies the UFM1 pathway to affect HCMV US2-mediated degradation of HLA class I

A

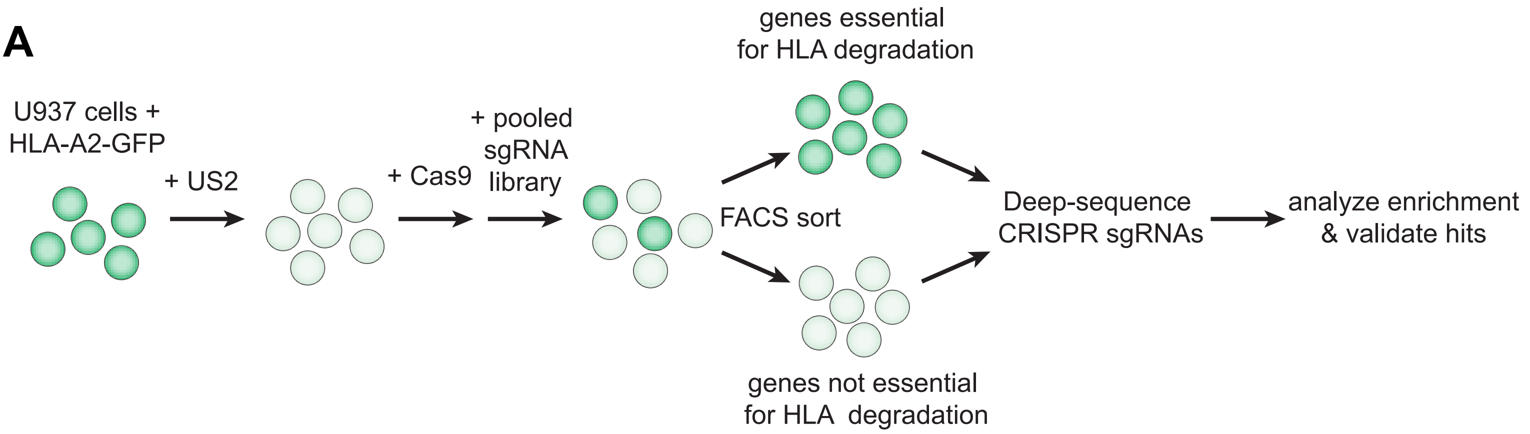

B

Gene ID	Gene symbol	Full name	Enriched at: 7 dpi	Enriched at: 18 dpi	Included in validation studies:
550	AUP1	Ancient Ubiquitous Protein 1		✓	
567	B2M	Beta-2 Microglobulin	✓	✓	
79587	CARS2	Cysteinyl-tRNA Synthetase 2, Mitochondrial		✓	✓
27235	COQ2	Coenzyme Q2, Polyprenyltransferase		✓	✓
23197	FAF2	Fas-Associated Factor Family Member 2	✓	✓	
192286	HIGD2A	HIG1 Hypoxia Inducible Domain Family Member 2A		✓	✓
6782	HSPA13	Heat Shock Protein Family (Hsp70) Member 13	✓		✓
4528	MTIF2	Mitochondrial Translation Initiation Factor 2		✓	✓
54539	NDUFB11	NADH:Ubiquinone Oxidoreductase Subunit B11		✓	✓
55666	NPLOC4	NPL4 Homolog, Ubiquitin Recognition Factor	✓	✓	
5687	PSMA6	Proteasome Subunit Alpha 6	✓		
5695	PSMB7	Proteasome Subunit Beta 7	✓		✓
5702	PSMC3	Proteasome 26S Subunit, ATPase 3	✓		
5705	PSMC5	Proteasome 26S Subunit, ATPase 5	✓		
5718	PSMD12	Proteasome 26S Subunit, Non-ATPase 12	✓		
5719	PSMD13	Proteasome 26S Subunit, Non-ATPase 13	✓		✓
5709	PSMD3	Proteasome 26S Subunit, Non-ATPase 3	✓		
254958	REXO1L1	RNA Exonuclease 1 Homolog Like 1, Pseudogene	✓		✓
11236	RNF139	Ring Finger Protein 139	✓	✓	✓
29927	SEC61A1	SEC61 Translocon Alpha 1 Subunit	✓	✓	✓
10952	SEC61B	SEC61 Translocon Beta Subunit		✓	✓
7095	SEC62	SEC62 Homolog, Preprotein Translocation Factor	✓	✓	✓
11231	SEC63	SEC63 Homolog, Protein Translocation Regulator		✓	✓
6727	SRP14	Signal Recognition Particle 14	✓		
6728	SRP19	Signal Recognition Particle 19	✓		
6731	SRP72	Signal Recognition Particle 72	✓		✓
6734	SRPR	SRP Receptor Alpha Subunit	✓		✓
58477	SRPRB	SRP Receptor Beta Subunit	✓		✓
90871	TMEM261	Distal Membrane Arm Assembly Complex 1		✓	✓
79876	UBA5	Ubiquitin Like Modifier Activating Enzyme 5		✓	✓
7327	UBE2G2	Ubiquitin Conjugating Enzyme E2 G2	✓	✓	
7353	UFD1L	Ubiquitin Fusion Degradation 1 Like	✓		
51569	UFM1	Ubiquitin Fold Modifier 1	✓		✓
7415	VCP	Valosin Containing Protein	✓		

Figure 1 (continued)

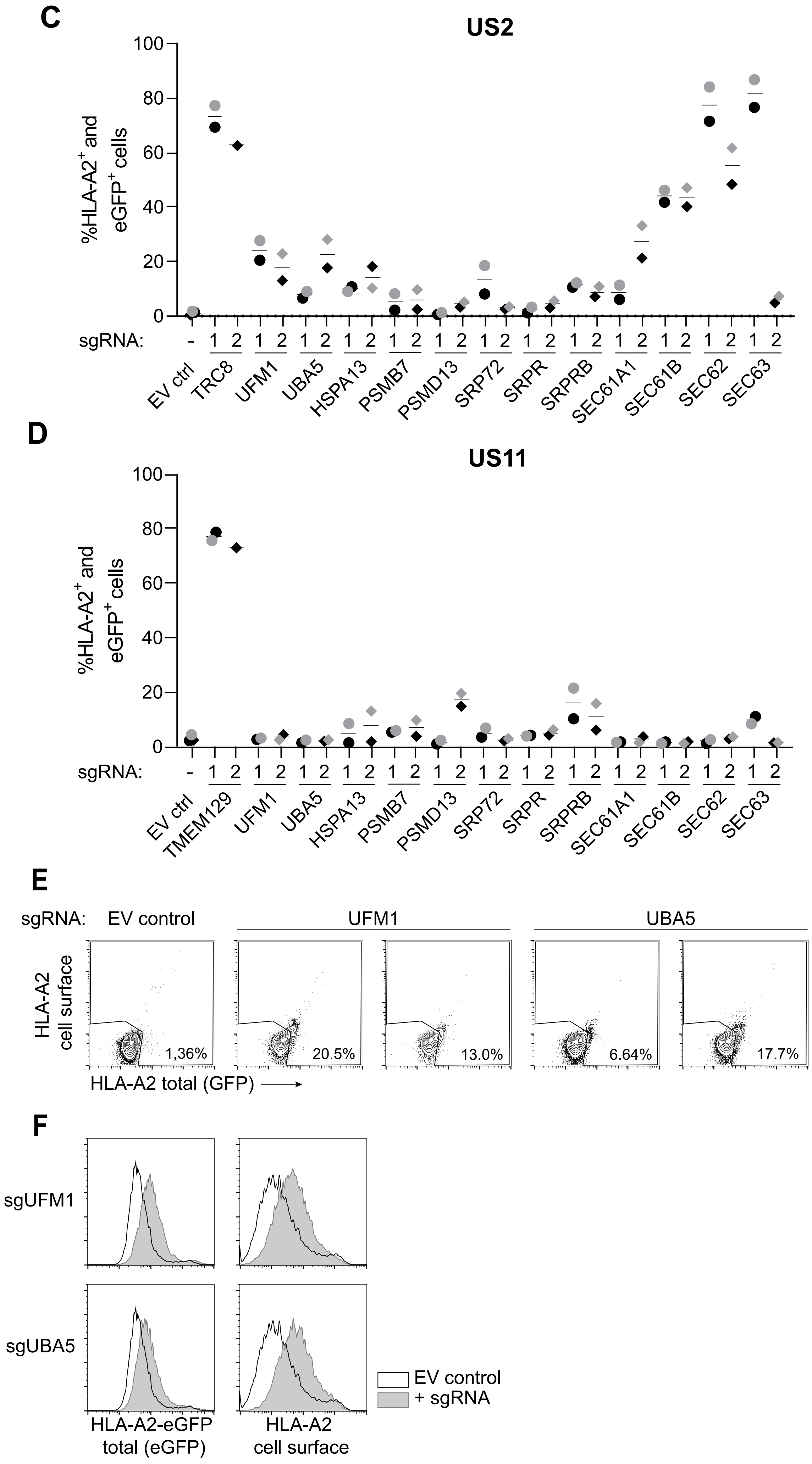

Figure 2: US2-mediated HLA class I expression is rescued upon knockout of multiple players in the UFMylation pathway

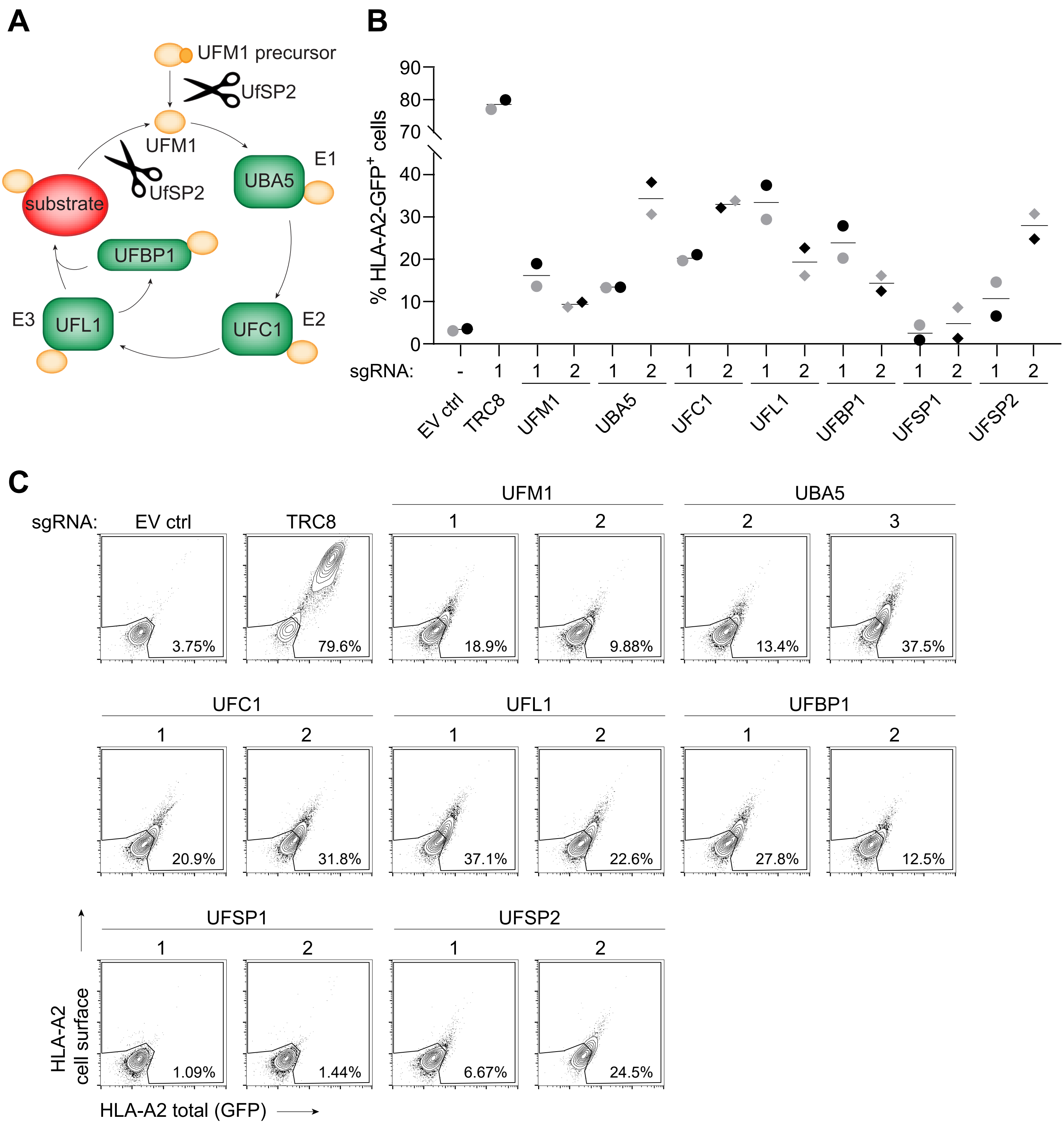

Figure 3: Clonal knockout cell lines for UFM1 and UBA5 show stable HLA-I rescue in the presence of HCMV US2

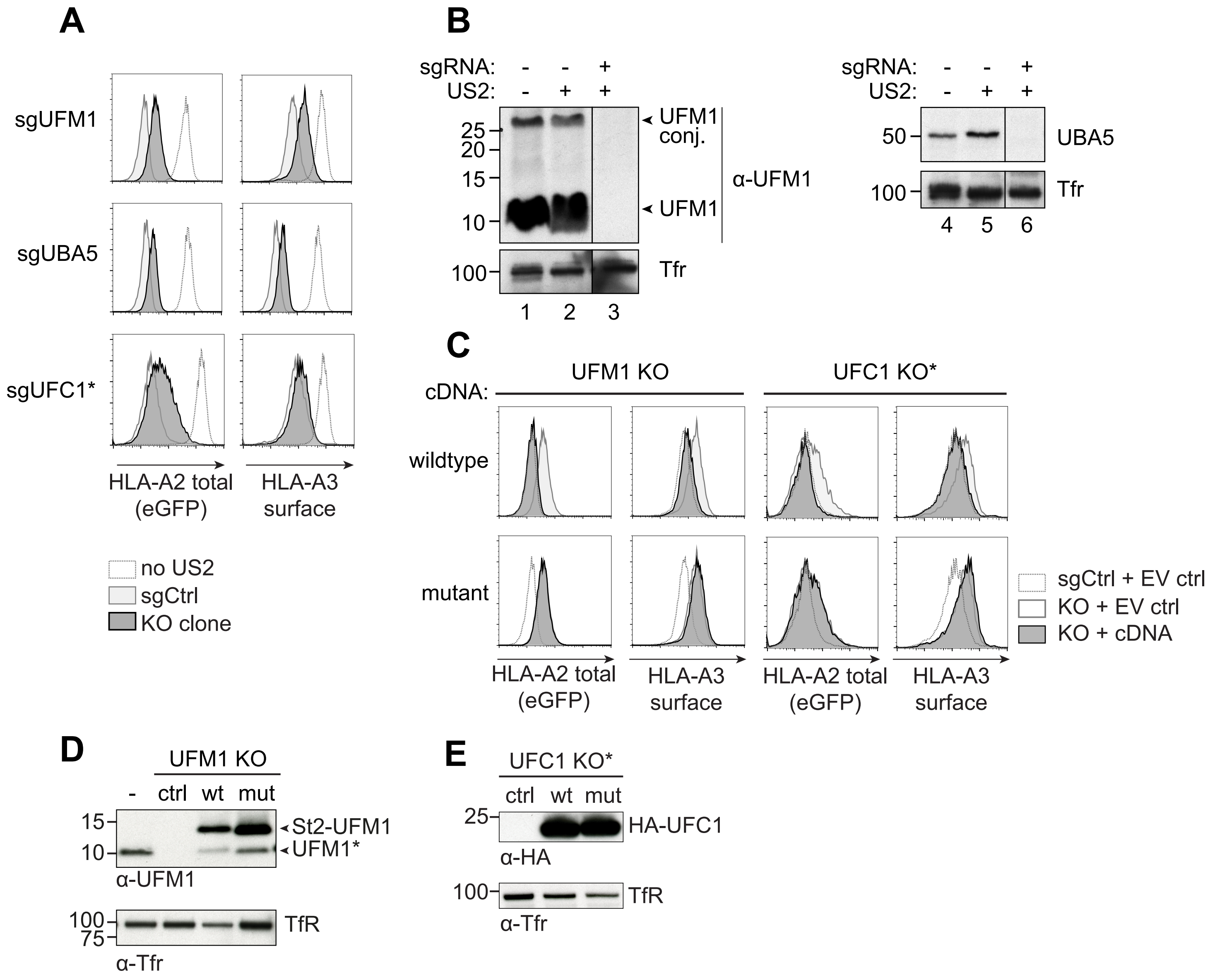

Figure 4: HLA class I, US2, and p97 are not UFMylated in US2-expressing cells

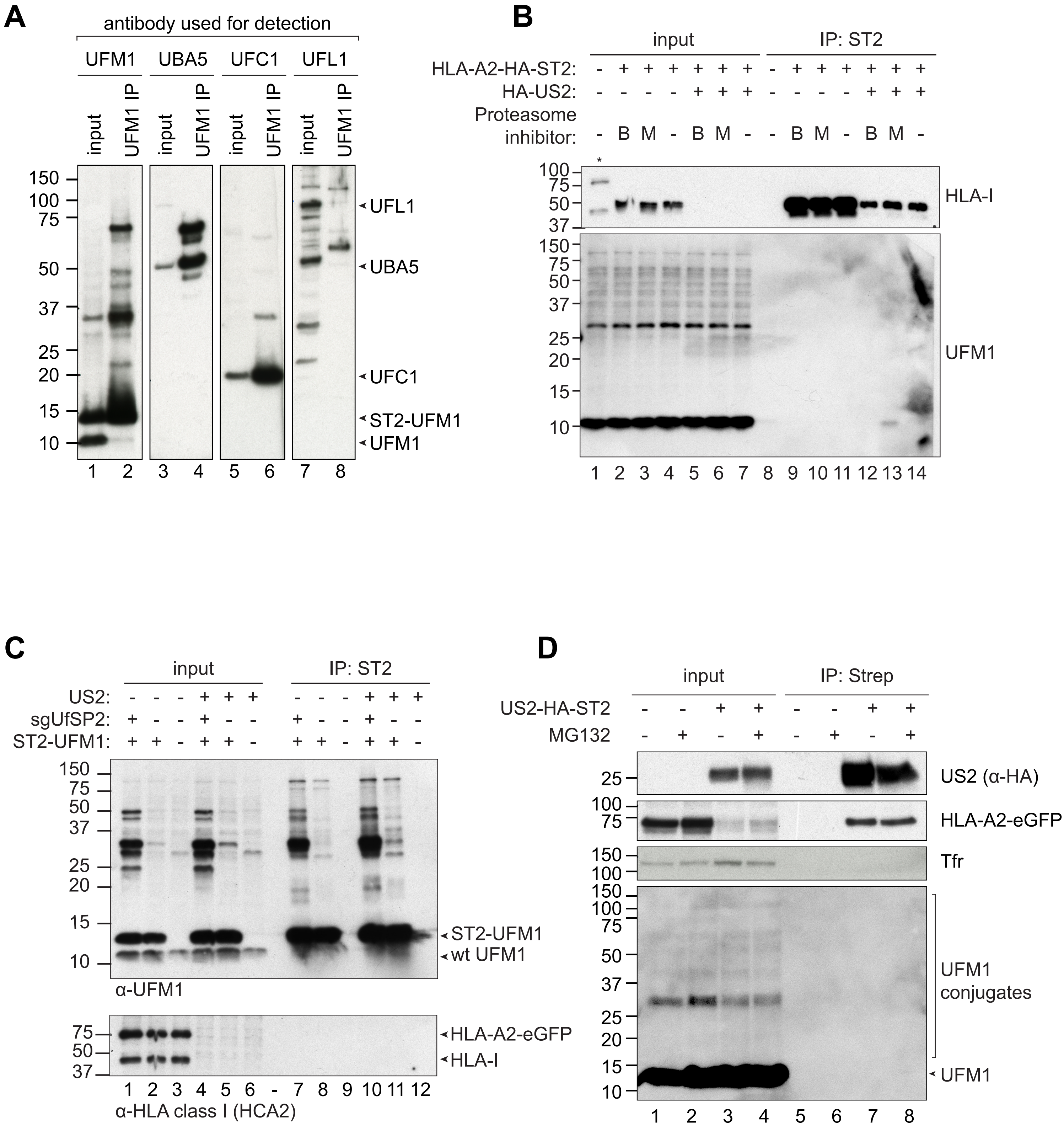

Figure 5: Mass spectrometry identifies ribosomal UFMylation in the presence of US2

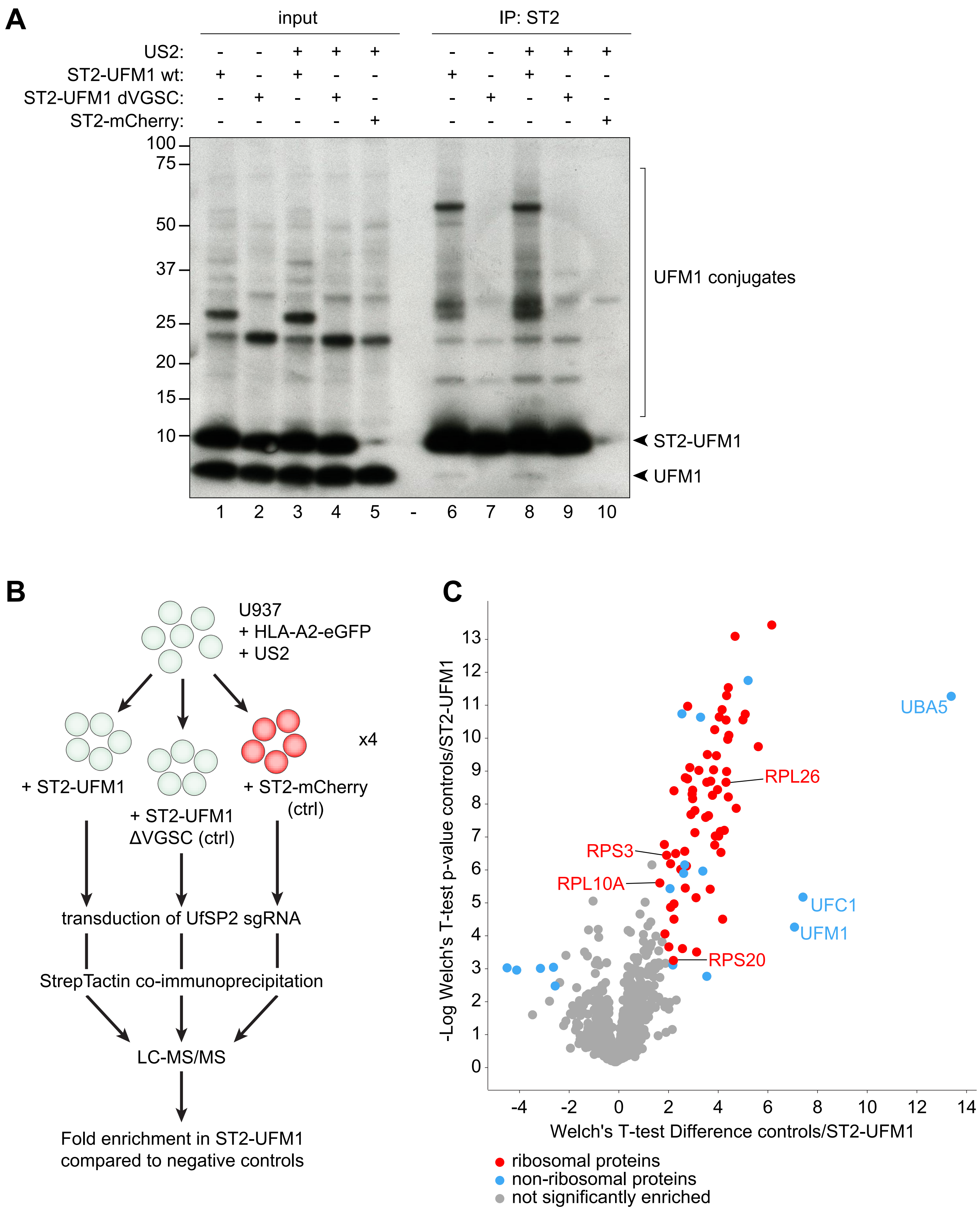

Figure 6: Functional UFM1 is required for efficient HLA class I dislocation to the cytosol

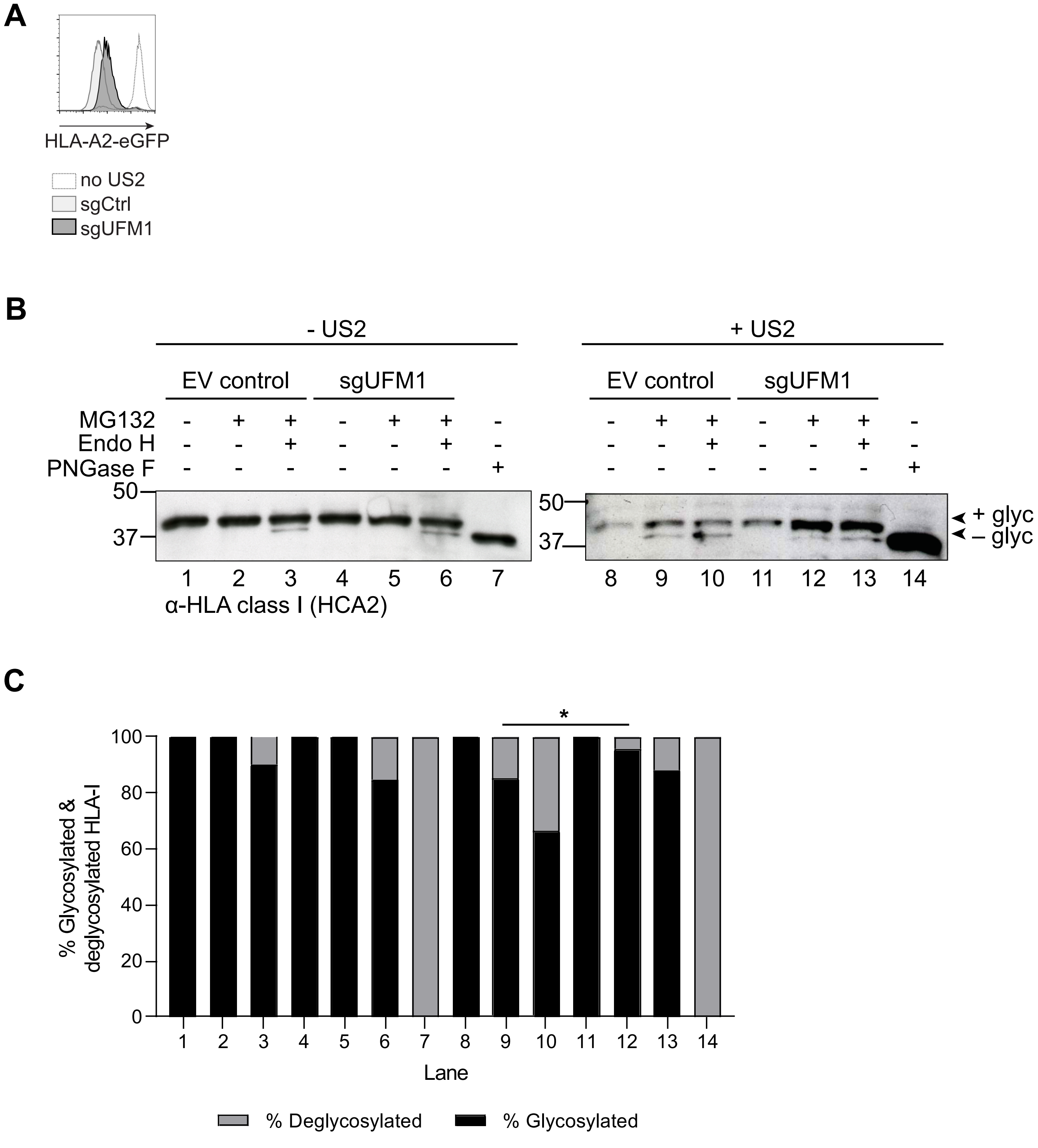

Supplement: Supplementary file 1 [file molecules-26-00287-s001.zip › molecules-1033577-supplementary and new figures/molecules-1033577-new figures.pdf]
