# Supplementary material for: The UFM1 Pathway Impacts HCMV US2-Mediated Degradation of HLA Class I
Source: Molecules. 2021 Jan 8;26(2):287. doi: 10.3390/molecules26020287 (PMC7827699; doi:10.3390/molecules26020287)
Supplement: Supplementary file 1 [file molecules-26-00287-s001.zip › molecules-1033577-supplementary and new figures/molecules-1033577-supplementary.pdf]

Supplementary figures

Figure S1: Validation of the genome-wide library screen at 7, 11, 18 and 28 days post-infection

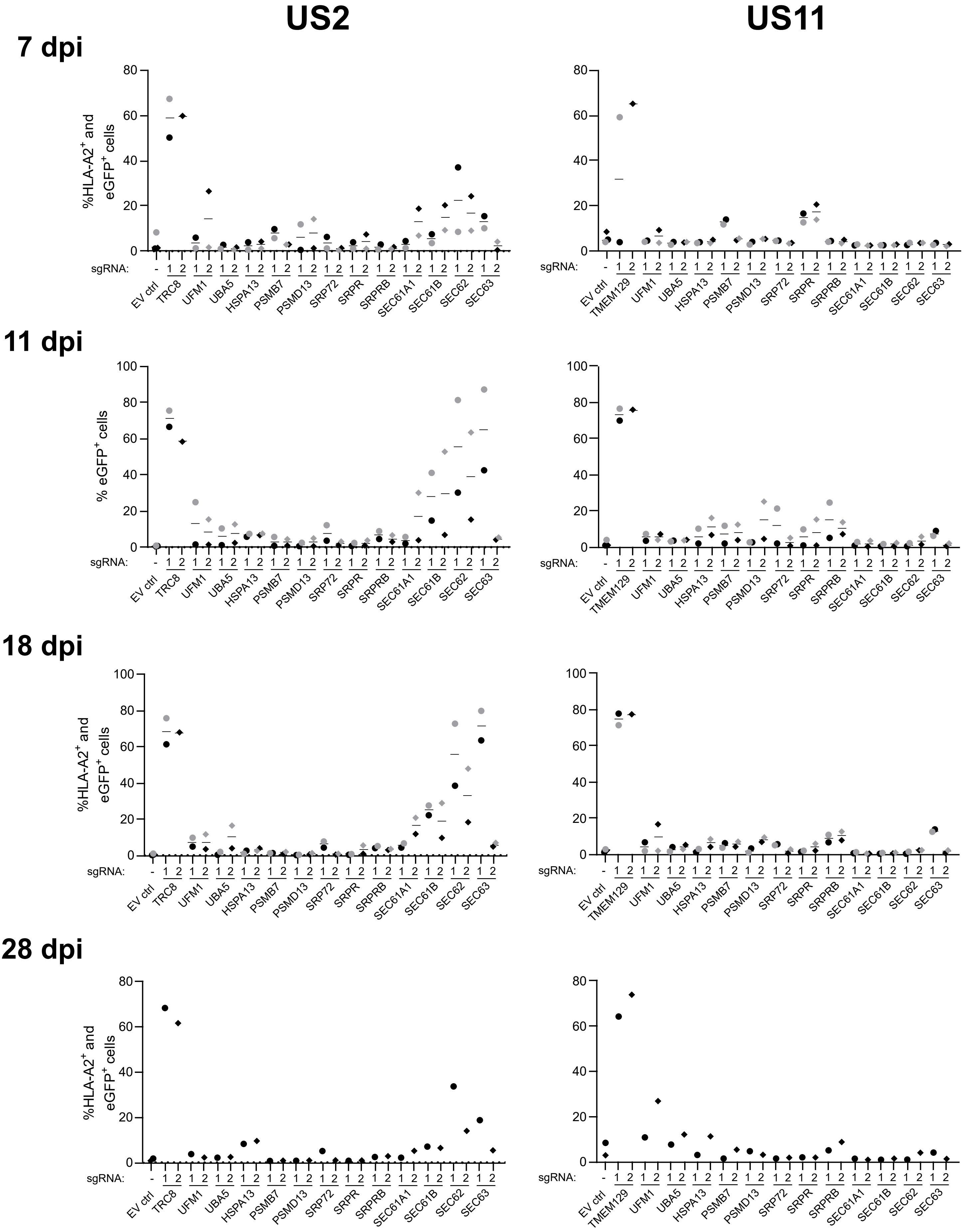

Figure S1: Validation of the genome-wide library screen at 7, 11, 18 and 28 days post-infection. Validation of the genome-wide screen was performed as described for Figure 1C.

Figure S2: Determination of the optimal timepoint for detecting HLA-I rescue

A

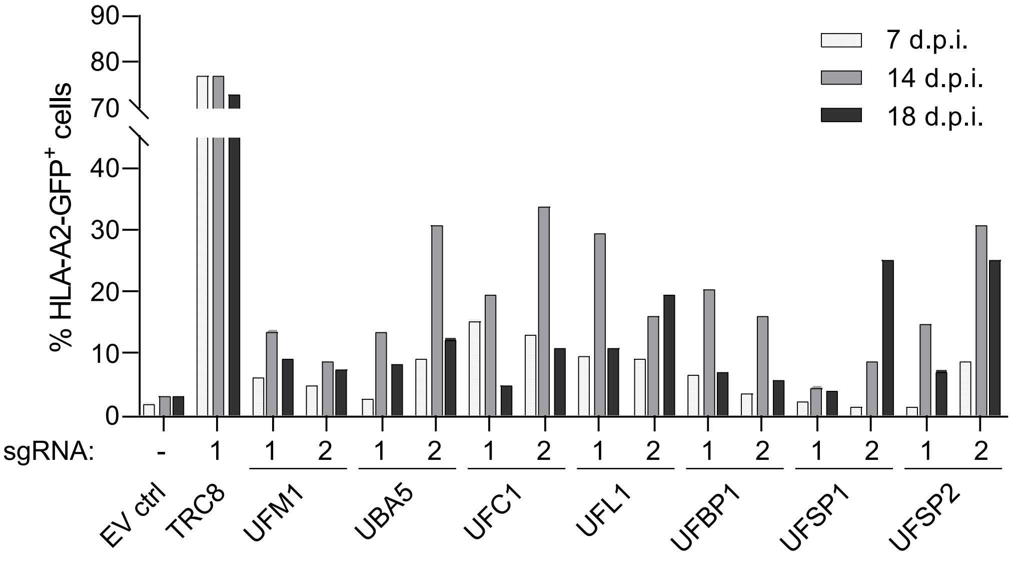

B

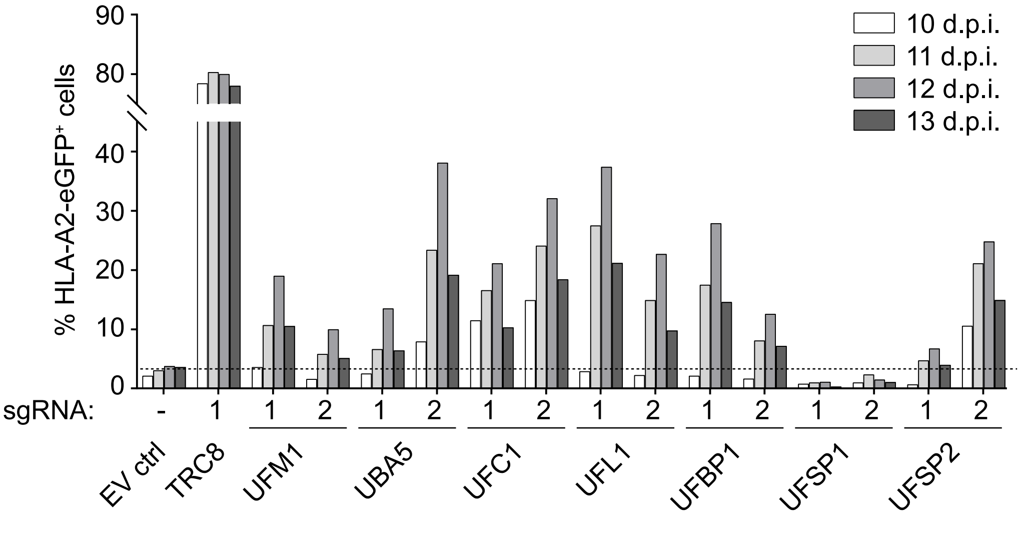

Figure S2: Determination of the optimal timepoint for detecting HLA-I rescue.
sgRNAs targeting TRC8 or all players of the UFMylation pathway were introduced as described in figure 2. Flow cytometry was performed on multiple days in two independent experiments **(A)** and **(B)** to determine the optimal timepoint for detecting HLA-A2-eGFP rescue. Figure 2B shows a selection of the data from these experiments: the 14 d.p.i. (in A) and 12 d.p.i. (in B) timepoints.

Figure S3: Clonal knockout cell lines for UFM1 and UBA5 show stable HLA-I rescue in the presence of HCMV US2

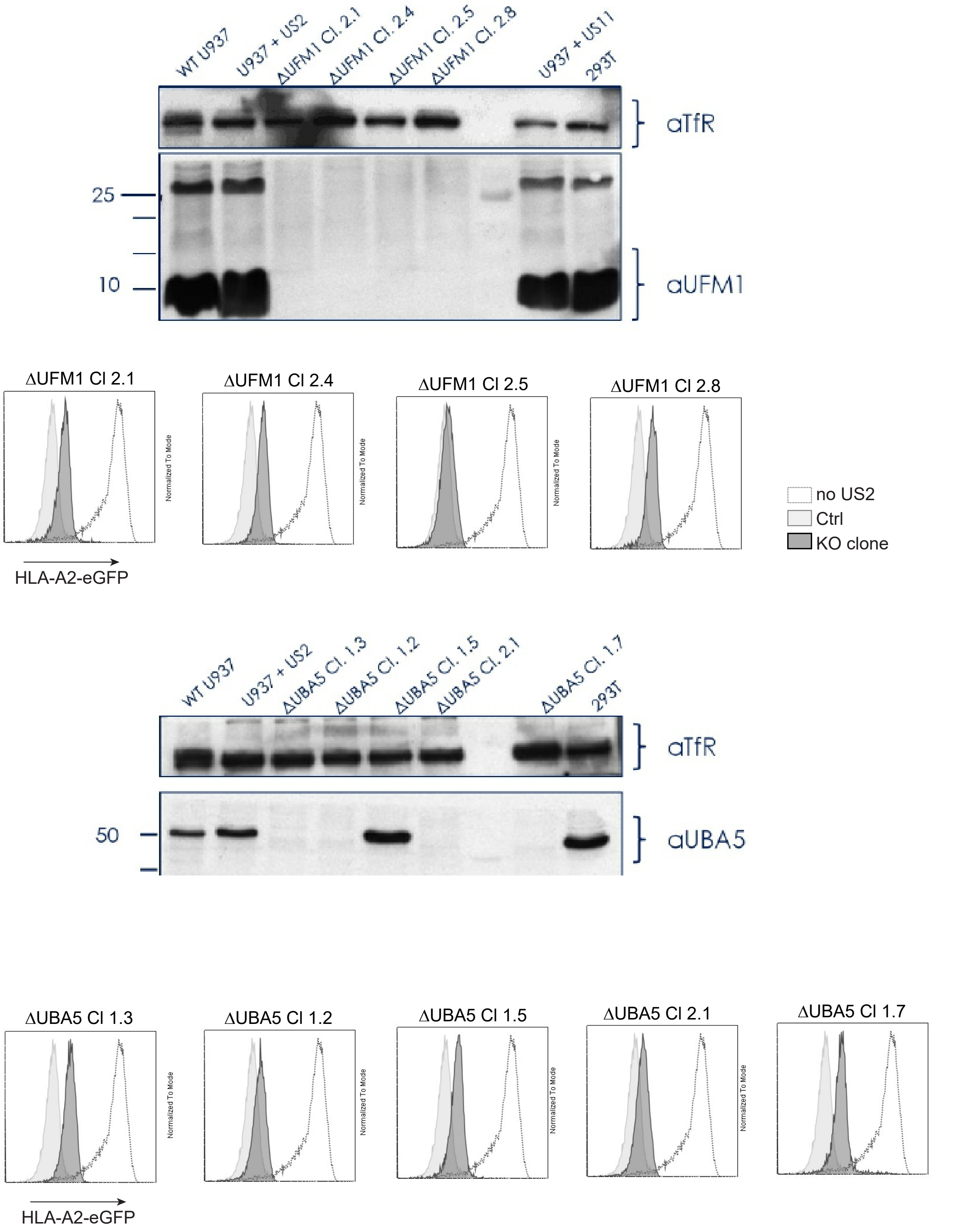

Figure S3. Clonal knockout cell lines for UFM1 and UBA5 show HLA-I rescue in the presence of HCMV US2. Western blot and flow cytometry analysis of additional clonal knockout cell lines for UFM1 and UBA5, as described in Figure 3. Note: the UBA5 clone 1.5 contains an in frame deletion (-15) and an out of frame deletion (-2); apparently, the mutant UBA5 protein encoded by the allele containing the in frame deletion is detectable but it is not functional. As a result, a rescue of HLA-I is observed.

Figure S4: Mutant UFM1 lacking its C-terminus is unable to bind UBA5

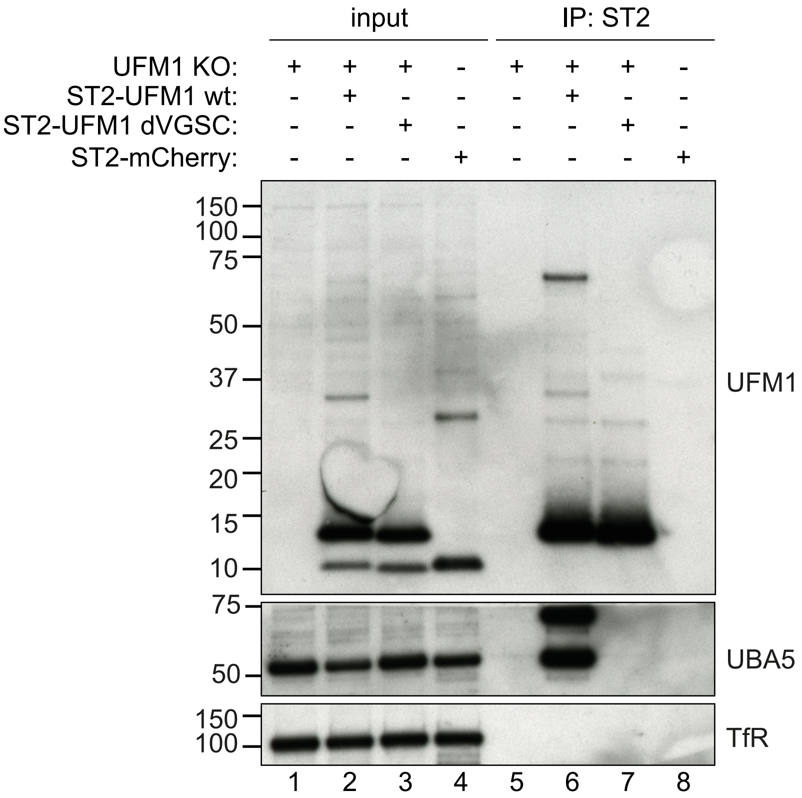

Figure S4: Mutant UFM1 lacking its C-terminus is unable to bind UBA5. StrepII-tagged WT UFM1 or a ΔVGSC mutant were expressed in clonal UFM1 knockout cells. As a control, StrepII-mCherry was introduced in UFM1-expressing cells. These StrepII-tagged constructs were immunoprecipitated in 1% LMNG lysis buffer. Input and immunoprecipitation samples were loaded on Western blot and stained for UFM1.

Figure S5: HLA class I is ubiquitinated in US2 expressing cells upon proteasome inhibition

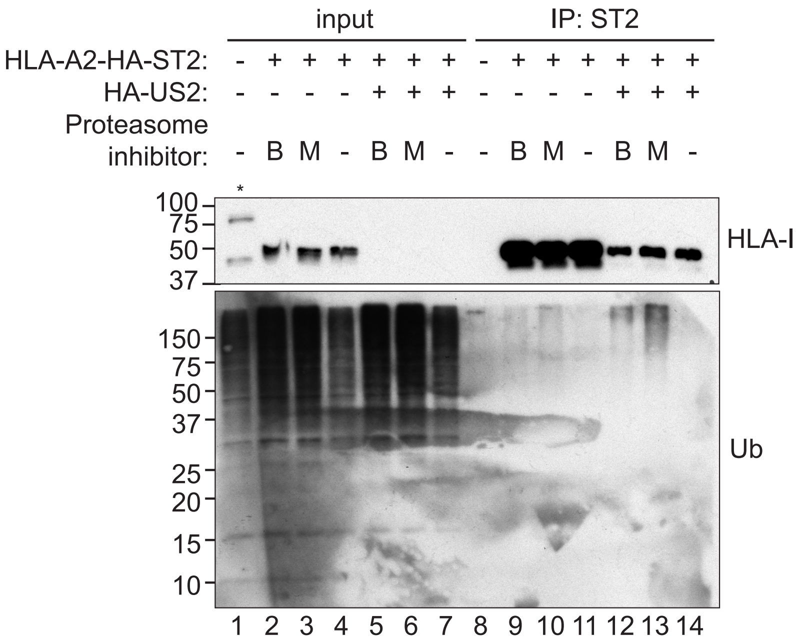

Figure S5: HLA class I is ubiquitinated in US2 expressing cells upon proteasome inhibition. U937 cells expressing HLAA2-HA-ST2 in the presence or absence of US2 were incubated with the proteasome inhibitors Bortezomib (B) or MG132 (M) to accumulate ubiquitinated HLA class I that would otherwise be degraded by the proteasome. HLA-A2-HA-StrepII was immunoprecipitated from these cells in 1% Digitonin lysis buffer, immunoblotted, and stained for ubiquitination.

Supplementary information 1

Target genes and genomic target sites of sgRNAs used in this study.

sgRNA name	sgRNA target site
TRC8_1	AGGAAGATGACAGGCGTCT
TMEM129_1	GCACACGGCGAACACCAGAT
UFM1_1	AAACGCACAGACTGCTGGTGAGTA
UFM1_2	ACCGACAAGTGCAATTATTACCAA
UBA5_1	ACCGAGTGTGATGACAGAAATTGC
UBA5_2	GCCTACTATTGCTACGGCAA
UFC1_1	CCCACAACTCACGATCTCG
UFC1_2	CGGTGCTGAAGACTAACGC
UFL1_1	GTTGGCGGCCGACTTCCAGC
UFL1_2	gAACCGCCTAATCTCTTCCC
UFBP1_1	GTGGCGCCTGTGTGGTACT
UFBP1_2	GTAGCGGCGGCTCTGCTAGT
UFSP1_1	GCTGCCTCGCTCACTTCGGA
UFSP1_2	GCCTCTGCCTCGCTCACTT
UFSP2_1	gTAGCTGAAAAGCCAAATCA
UFSP2_2	GCTAGCTACTCCTAATGGT
COQ2_1	AGCTCACCCAAGGCTAGTTG
COQ2_2	GAATAGCTCCAGTGCCAAAG
REXO1L1_1	TGCGAGCCACAGCTCCCTGC
REXO1L1_2	CTATCTCCTTCAGTTCTGCT
HIGD2A_1	CACTTACCTATGGGTACCAC
HIGD2A_2	CTCAGCTCATGATGCGCACC
TMEM261_1	GCCGATGACCATCTGCGTAA
TMEM261_2	TCCATGGACCATTACGCAGA
CARS2_1	TTCCCTCGCCAGTCTTTATG
CARS2_2	TATGCTGCATCCAAAAACCT
MTIF2_1	GTTAAAGGGCGATAATCTGA
MTIF2_2	TCTATTACTGTTCCTTCCAC
NDUFB11_1	TCACAAGCCTCTCAGCTTCG
NDUFB11_2	TGGCCTATCTGCCTGACTAC
HSPA13_1	GATGACCATCGCGTGAACAG
HSPA13_2	CCAAGTCTATCACCAAGACG
PSMB7_1	CCAGCTCATTTCTTCCAACC
PSMB7_2	TAATAGGATGGCATAGTTCT
PSMD13_1	CCGCAGAGCATCTTTGTAGT
PSMD13_2	AATTCAGTTGTTGTGCCTCA
SRP72_1	AACTGCCCTGCATTGTAAAG
SRP72_2	TAACTCTCTCTCCTTTGAAA
SRPR_1	ACTGGTTTGCTGGTAGCCAA
SRPR_2	CTCAAAACTCTACCAAACCT
SRPRB_1	GGAAAACGTTGCTCTTTGTC
SRPRB_2	TACAGCACAGCTGTCAGTAA
SEC61A1_1	TGTTGTACTGGCCACGGTAG
SEC61A1_2	ATCAAGTCGGCCCGCTACCG
SEC61B_1	TAGTGGCCCTGTTCCAGTAT
SEC61B_2	GTAGAATCGCCACATCCCCC
SEC62_1	CTGTGGTTGACTACTGCAAC
SEC62_2	GTAGTCAACCACAGACTCCC
SEC63_1	GTGATGAGGTTATGTTCATG
SEC63_2	TTGGTATTCTCGGTCTGTTT

Supplementary information 2

Depleted

UniProtKB	Gene name	Full name	Function
P12814	**ACTN1**	Actinin Alpha 1	Actin-binding protein
Q13155	**AIMP2**	Aminoacyl tRNA Synthetase Complex Interacting Multifunctional Protein 2	Non-enzymatic factor of aminoacyl tRNA-synthetase complex
P01344	**IGF2**	Insulin-Like Growth Factor 2	Insulin-like growth factor (imprinted gene related to tumorigenesis)
O95613	**PCNT**	Pericentrin	Integral component of the pericentiolar material; binds to calmodulin
Q9H4A3	**WNK1**	WNK Lysine Deficient Protein Kinase 1	Regulation of electrolyte homeostasis

Enriched non-ribosomal proteins (blue)

UniProtKB	Gene name	Full name	Function
P47813	**EIF1AX**	Eukaryotic Translation Initiation Factor 1A, X-linked	Binding of 40S ribosome, eIF2, GTP, Met-tRNAi and eIF3 to 5' end of capped mRNA
P62861	**FAU**	Ubiquitin Like and Ribosomal Protein S30 Fusion	Fusion protein of ubiquitin-like protein FUBI (N-term) and ribosomal protein S30 (C-term)
P63244	**GNB2L1**	Guanidine Nucleotide Binding Protein (G-protein), Beta Polypeptide 2-Like	Scaffolding protein in 40S ribosomal subunit involved in ribosomal quality control
P19338	**NCL**	Nucleolin	Nucleolar phosphoprotein involved in synthesis and maturation of ribosomes
Q96S44	**TP53RK**	TP53 Regulating Kinase	Required for the modification of tRNAs that read codons beginning with alanine
P62995	**TRA2B**	Transformer 2 Beta Homolog	Nuclear protein involved in pre-mRNA splicing
Q8WZ42	**TTN**	Titin	Key component in the assembly and functioning of vertebrate striated muscle
O43396	**TXNL1**	Thioredoxin Like 1	Active thioredoxin; component of the 19S regulatory cap of the 26S proteasome
Q9GZZ9	**UBA5**	Ubiquitin Like Modifier Activating Enzyme 5	E1 activating enzyme for UFM1
Q9Y3C8	**UFC1**	Ubiquitin-Fold Modifier Conjugating Enzyme 1	E2 conjugating enzyme for UFM1
P61960	**UFM1**	Ubiquitin Fold Modifier 1	Ubiquitin-like molecule that is conjugated to target proteins in a manner analogous to ubiquitination
Q92900	**UPF1**	Up-Frameshift Mutation 1 Homolog	nonsense mediated decay of mRNAs; recruited to mRNAs upon translation termination and to stalled ribosomes

Enriched ribosomal proteins (red)

UniProtKB	Gene name	Full name	Function
P62906	**RPL10A**	Ribosomal Protein L10a	ribosomal protein that is a component of the 60S subunit
P62913	**RPL11**	Ribosomal Protein L11	ribosomal protein that is a component of the 60S subunit
P30050	**RPL12**	Ribosomal Protein L12	ribosomal protein that is a component of the 60S subunit
P26373	**RPL13**	Ribosomal Protein L13	ribosomal protein that is a component of the 60S subunit
P40429	**RPL13A**	Ribosomal Protein L13A	ribosomal protein that is a component of the 60S subunit
P50914	**RPL14**	Ribosomal Protein L14	ribosomal protein that is a component of the 60S subunit
P61313	**RPL15**	Ribosomal Protein L15	ribosomal protein that is a component of the 60S subunit
P18621	**RPL17**	Ribosomal Protein L17	ribosomal protein that is a component of the 60S subunit
Q07020	**RPL18**	Ribosomal Protein L18	ribosomal protein that is a component of the 60S subunit
Q02543	**RPL18A**	Ribosomal Protein L18A	ribosomal protein that is a component of the 60S subunit
P84098	**RPL19**	Ribosomal Protein L19	ribosomal protein that is a component of the 60S subunit
P46778	**RPL21**	Ribosomal Protein L21	ribosomal protein that is a component of the 60S subunit
P62750	**RPL23A**	Ribosomal Protein L23A	ribosomal protein that is a component of the 60S subunit
P83731	**RPL24**	Ribosomal Protein L24	ribosomal protein that is a component of the 60S subunit
P61254	**RPL26**	Ribosomal Protein L26	ribosomal protein that is a component of the 60S subunit
P61353	**RPL27**	Ribosomal Protein L27	ribosomal protein that is a component of the 60S subunit
P46776	**RPL27A**	Ribosomal Protein L27A	ribosomal protein that is a component of the 60S subunit
P46779	**RPL28**	Ribosomal Protein L28	ribosomal protein that is a component of the 60S subunit
P47914	**RPL29**	Ribosomal Protein L29	ribosomal protein that is a component of the 60S subunit

P39023	**RPL3**	Ribosomal Protein L3	ribosomal protein that is a component of the 60S subunit
P62899	**RPL31**	Ribosomal Protein L31	ribosomal protein that is a component of the 60S subunit
P62910	**RPL32**	Ribosomal Protein L32	ribosomal protein that is a component of the 60S subunit
P49207	**RPL34**	Ribosomal Protein L34	ribosomal protein that is a component of the 60S subunit
P42766	**RPL35**	Ribosomal Protein L35	ribosomal protein that is a component of the 60S subunit
P18077	**RPL35A**	Ribosomal Protein L35A	ribosomal protein that is a component of the 60S subunit
Q9Y3U8	**RPL36**	Ribosomal Protein L36	ribosomal protein that is a component of the 60S subunit
P61513	**RPL37A**	Ribosomal Protein L37A	ribosomal protein that is a component of the 60S subunit
P62891	**RPL39**	Ribosomal Protein L39	ribosomal protein that is a component of the 60S subunit
P36578	**RPL4**	Ribosomal Protein L4	ribosomal protein that is a component of the 60S subunit
P46777	**RPL5**	Ribosomal Protein L5	ribosomal protein that is a component of the 60S subunit
Q02878	**RPL6**	Ribosomal Protein L6	ribosomal protein that is a component of the 60S subunit
P18124	**RPL7**	Ribosomal Protein L7	ribosomal protein that is a component of the 60S subunit
P62424	**RPL7A**	Ribosomal Protein L7A	ribosomal protein that is a component of the 60S subunit
P62917	**RPL8**	Ribosomal Protein L8	ribosomal protein that is a component of the 60S subunit
P32969	**RPL9**	Ribosomal Protein L9	ribosomal protein that is a component of the 60S subunit
P05388	**RPLP0**	Ribosomal Protein Lateral Stalk Subunit P0	ribosomal protein that is a component of the 60S subunit
P05386	**RPLP1**	Ribosomal Protein Lateral Stalk Subunit P1	ribosomal protein that is a component of the 60S subunit
P05387	**RPLP2**	Ribosomal Protein Lateral Stalk Subunit P2	ribosomal protein that is a component of the 60S subunit
P46783	**RPS10**	Ribosomal Protein S10	ribosomal protein that is a component of the 40S subunit
P62280	**RPS11**	Ribosomal Protein S11	ribosomal protein that is a component of the 40S subunit
P62277	**RPS13**	Ribosomal Protein S13	ribosomal protein that is a component of the 40S subunit
P62263	**RPS14**	Ribosomal Protein S14	ribosomal protein that is a component of the 40S subunit
P62841	**RPS15**	Ribosomal Protein S15	ribosomal protein that is a component of the 40S subunit
P62249	**RPS16**	Ribosomal Protein S16	ribosomal protein that is a component of the 40S subunit
P08708	**RPS17**	Ribosomal Protein S17	ribosomal protein that is a component of the 40S subunit
P39019	**RPS19**	Ribosomal Protein S19	ribosomal protein that is a component of the 40S subunit
P15880	**RPS2**	Ribosomal Protein S2	ribosomal protein that is a component of the 40S subunit
P60866	**RPS20**	Ribosomal Protein S20	ribosomal protein that is a component of the 40S subunit
P63220	**RPS21**	Ribosomal Protein S21	ribosomal protein that is a component of the 40S subunit
P62266	**RPS23**	Ribosomal Protein S23	ribosomal protein that is a component of the 40S subunit
P62847	**RPS24**	Ribosomal Protein S24	ribosomal protein that is a component of the 40S subunit
P62851	**RPS25**	Ribosomal Protein S25	ribosomal protein that is a component of the 40S subunit
P62854	**RPS26**	Ribosomal Protein S26	ribosomal protein that is a component of the 40S subunit
Q71UM5	**RPS27L**	Ribosomal Protein S27L	ribosomal protein that is a component of the 40S subunit
P62857	**RPS28**	Ribosomal Protein S28	ribosomal protein that is a component of the 40S subunit
P23396	**RPS3**	Ribosomal Protein S3	ribosomal protein that is a component of the 40S subunit
P61247	**RPS3A**	Ribosomal Protein S3A	ribosomal protein that is a component of the 40S subunit
P62701	**RPS4X**	Ribosomal Protein S4X	ribosomal protein that is a component of the 40S subunit
P46782	**RPS5**	Ribosomal Protein S5	ribosomal protein that is a component of the 40S subunit
P62753	**RPS6**	Ribosomal Protein S6	ribosomal protein that is a component of the 40S subunit
P62081	**RPS7**	Ribosomal Protein S7	ribosomal protein that is a component of the 40S subunit
P62241	**RPS8**	Ribosomal Protein S8	ribosomal protein that is a component of the 40S subunit
P46781	**RPS9**	Ribosomal Protein S9	ribosomal protein that is a component of the 40S subunit
P08865	**RPSA**	Ribosomal Protein SA	Required for the assembly and/or stability of the 40S ribosomal subunit and for the processing of the 20S rRNA-precursor to mature 18S rRNA

List of significantly enriched/depleted UFM1-binding proteins: Overview of gene names and protein functions of the hits identified in mass spectrometric analysis of proteins interacting with UFM1. (In Figure 5C, only significantly enriched proteins (red and blue) are shown.).
